# YAP1 Enhances Mesenchymal-Type Gene Expression in Human Adrenergic-Type Neuroblastoma Cells

**DOI:** 10.3390/cancers18030383

**Published:** 2026-01-26

**Authors:** Marius Ludwig, Kerstin Ahrens, Annika Winkler, Jasmin Wünschel, Peris Ruka, Marco Lodrini, Falk Hertwig, Sveva Castelli, Theresa M. Thole-Kliesch, Jan F. Hollander, Steffen Fuchs, Annette Künkele, Marvin Jens, Soulafa Mamlouk, Steven W. Warmann, Kathy Astrahantseff, Angelika Eggert, Johannes H. Schulte, Annabell Szymansky, Hedwig E. Deubzer

**Affiliations:** 1 Department of Pediatric Oncology and Hematology, Charité-Universitätsmedizin Berlin, Corporate Member of Freie Universität Berlin and Humboldt-Universität zu Berlin, Campus Virchow Klinikum, 13353 Berlin, Germany; marius.ludwig@charite.de (M.L.); annika.winkler@dhzc-charite.de (A.W.); jasmin.wuenschel@charite.de (J.W.); peris.ruka@charite.de (P.R.); marco.lordini@charite.de (M.L.); sveva.castelli@charite.de (S.C.); theresa.thole-kliesch@charite.de (T.M.T.-K.); jan-fredrick.hollander@charite.de (J.F.H.); steffen.fuchs@charite.de (S.F.); annette.kuenkele@charite.de (A.K.); marvin.jens@charite.de (M.J.); kathy.astrahantseff@charite.de (K.A.); annabell.szymansky@charite.de (A.S.); 2 Berlin Institute of Health (BIH) at Charité, 10178 Berlin, Germany; 3 German Cancer Consortium (DKTK), Partner Sites Berlin and Tübingen, German Cancer Research Center (DKFZ), 69120 Heidelberg, Germany; 4 Department of Pediatric Surgery, Charité-Universitätsmedizin Berlin, Corporate Member of Freie Universität Berlin and Humboldt-Universität zu Berlin, Campus Virchow Klinikum, 13353 Berlin, Germany; soulafa.mamlouk@charite.de (S.M.); steven.warmann@charite.de (S.W.W.); 5 University Hospital Essen, 45147 Essen, Germany; angelika.eggert@uk-essen.de; 6 Department of Pediatric Oncology and Hematology, University Hospital Tübingen, 72076 Tübingen, Germany; johannes.schulte@med.uni-tuebingen.de; 7 Experimental and Clinical Research Center (ECRC) of Charité and Max-Delbrück-Center of Molecular Medicine in the Helmholtz Association, Lindenberger Weg 80, 13125 Berlin, Germany

**Keywords:** pediatric cancer, embryonal tumor, phenotypic plasticity, drug resistance, relapse, RNA sequencing, gene expression signature

## Abstract

**Background/Objectives**: Neuroblastoma cells are phenotypically plastic, transitioning between mesenchymal and adrenergic states. Core functional genes (e.g., *YAP1*) mark the mesenchymal state, which is linked to unfavorable prognosis. We and others previously demonstrated relapse-specific Hippo-YAP pathway activation in matched primary/relapsed neuroblastomas. Here we explored the role of *YAP1* in neuroblastoma aggressiveness and response to therapy. **Methods**: RT-qPCR and immunoblotting assessed *YAP1* expression in neuroblastoma cell lines. RNA-sequencing detected *YAP1*-dependent gene signatures in Tet-ON SK-N-AS and SH-EP neuroblastoma cell models expressing wildtype YAP1 or constitutively activated YAP1^S127A^. Data from cell models were compared with our published *YAP1* expression data from neuroblastomas. Efficacy of commonly used chemotherapeutics was comparatively analyzed in the cell models. **Results**: YAP1 expression showed marked variability across a panel of neuroblastoma cell lines, assessed by mRNA analysis in 10 cell lines and protein analysis in a subset of 9 cell lines. RNA sequencing in constitutively activated YAP1^S127A^ mutant and wildtype YAP1 models detected 2162 and 1837 significantly differentially expressed genes in the SK-N-AS and SH-EP backgrounds, respectively. Continuously activating YAP1 in SK-N-AS cells upregulated mesenchymal signature genes and mesenchymal-associated transcription factors. Gene expression influenced by YAP1 activity in the cell models significantly overlapped with YAP1-associated genes (e.g., *CYR61* and *SPRY4*) in published tumor data. Functionally, *YAP1^S127A^* expression rendered neuroblastoma cells resistant to chemotherapy. **Conclusions**: Findings corroborate the idea of a mechanistic role for *YAP1* in neuroblastoma adrenergic to mesenchymal reprogramming and therapy resistance. The *YAP1*-mediated plastic switch towards a mesenchymal expression state in neuroblastoma cells may provide the molecular basis for novel therapeutic avenues.

## Simple Summary

More than half of infants and children with high-risk neuroblastoma will experience relapse despite intensive therapy that includes multiple chemotherapy agents. Survival is as low as 20% after relapse. To improve survival, we must better understand why these tumors are so resistant to treatment, often even before prior exposure to chemotherapy. Molecular studies of relapsed tumors indicate that neuroblastoma cells are capable of varying their nature and capabilities without altering the genome. The mesenchymal cell form was strongly enriched in relapsed tumors. We show here that the YAP1 protein plays a role in shifting the neuroblastoma cell state towards the more difficult-to-treat mesenchymal form. We performed functional experiments and identified a profile of genes active when YAP1 is acting in neuroblastoma cell models and confirmed this profile in patient tumor samples. Our results help to better understand a facet of high-risk neuroblastoma that helps evade chemotherapy.

## 1. Introduction

Neuroblastoma is the most common extracranial solid tumor in children, contributing to up to 12% of childhood cancer deaths [1]. Half the patients with high-risk disease experience relapse, with <20% overall survival after first relapse [2,3]. Well-designed clinical umbrella trials with clearly structured therapy phases for patients with a first relapse of high-risk neuroblastoma (re-induction, consolidation, maintenance) are urgently needed. While first relapse is not yet a palliative setting, the emergence and further evolution of chemotherapy-resistant neuroblastoma clones at relapse present a major challenge for the clinical management of these patients [4,5].

YAP1 activation was associated with relapsed neuroblastoma in a study of paired primary and relapsed tumor samples from the same patients [6]. YAP1 is a transcriptional coactivator and one of the main downstream effectors of the Hippo pathway, a highly conserved signaling pathway playing essential roles in regulating organ size, cell death, and proliferation [7,8,9]. Hippo pathway proteins phosphorylate YAP1 and its paralog, TAZ, to negatively regulate their functions by cytoplasmic retention and degradation [10]. Unphosphorylated YAP1 translocates to the nucleus and binds to transcription factors of the TEA domain family to regulate target gene expression [11,12]. YAP1 has been shown to upregulate transcriptional programs associated with epithelial-to-mesenchymal transition in several cancers [13,14,15,16]. Increased YAP1 activity has been reported to induce a neural crest-like phenotype in SH-SY-5Y neuroblastoma cells, suggesting YAP1 may be able to shift neuroblastoma cell states [17].

Super-enhancer-driven phenotypic plasticity has been reported in neuroblastoma cells [18,19]. Transdifferentiation between mesenchymal (MES) and adrenergic (ADRN) cell states presents an alternative mechanism for therapy resistance and relapse to the selection of genetically distinct clones by therapeutic pressure [20,21]. MES cells were shown to be enriched in post-treatment and relapse samples [19,21], suggesting a potential role in therapy resistance and relapse. ADRN cells express genes active in the adrenal gland developmental lineage, including *PHOX2B*, *PHOX2A* and *GATA3*. MES neuroblastoma cells only express low levels of ADRN genes, but highly express a MES gene signature that includes *SNAI2*, *VIM* and *YAP1* [19]. MES cells were identified in primary neuroblastomas by Patel et al. [22], in contrast to previous studies performing single-cell RNA sequencing in tumor samples [23,24]. This emerging evidence corroborates the idea that MES cells could be clinically important, and not only an artifact of cell culture. Even though the PRRX1 and NOTCH transcription factors have been reported to induce transdifferentiation towards the MES state in neuroblastoma cells, the molecular mechanisms driving transdifferentiation remain incompletely understood [19,20].

By contributing to transdifferentiation between MES and ADRN phenotypes, YAP1 may contribute to patient mortality by opening paths to therapy resistance. How different transcription factors work together to orchestrate phenotypic plasticity remains incompletely understood. We generated inducible models with enhanced YAP1 activity by expressing the constitutively activated YAP1^S127A^ mutant in both a MES and ADRN cellular background provided by different neuroblastoma cell lines. The transcriptional effects of enhancing YAP1 activity were analyzed using RNA sequencing, and resilience to standard-of-care chemotherapy agents was used as a functional measure of MES-directed transdifferentiation. Data from our cell models were compared with published transcriptomes from patient tumor samples to assess the clinical significance of our findings. Here, we present evidence that increasing YAP1 activity induces the expression of MES genes in a neuroblastoma ADRN cell background and makes neuroblastoma cells more resilient to chemotherapy.

## 2. Materials and Methods

### 2.1. Cell Lines

The CHP134 (RRID: CVCL_1124), IMR-32 (RRID: CVCL_0346), Kelly (RRID: CVCL_2092), NGP (RRID: CVCL_2141), SH-EP (RRID: CVCL_0524), SK-N-AS (RRID: CVCL_1700), SK-N-BE (RRID: CVCL_0528), SK-N-FI (RRID: CVCL_1702) and SK-N-SH (RRID: CVCL_D044) neuroblastoma cell lines and the YAP1-expressing human control cell lines, DAOY (RRID: CVCL_1167), HEK293 (RRID: CVCL_0045), HeLa (RRID: CVCL_0030) and VH7 (primary human foreskin fibroblasts), were cultured in RPMI 1640 or DMEM medium (Thermo Fisher Scientific, Waltham, MA, USA) supplemented with 10% fetal calf serum (GE Healthcare) and 1% penicillin-streptomycin solution (10,000 U/mL penicillin, 10 mg/mL streptomycin) and maintained at 37 °C and 5% CO_2_. Cell lines were authenticated by high-throughput SNP-based assays (IDEXX Bioresearch, Westbrook, ME, USA; Multiplexion, Heidelberg, Germany) [25], and regularly monitored for *Acholeplasma laidlawii*, mycoplasma species, and squirrel monkey retrovirus infections using high-throughput, multiplexed testing [26].

### 2.2. Cell Model Generation with Enhanced YAP1 Expression

YAP1-enhanced cell models were generated using a tetracycline-inducible system [27] to express the constitutively activated YAP1^S127A^ mutant in the SK-N-AS and SH-EP neuroblastoma cell lines. Tet-repressor (TR)-harboring neuroblastoma cell models (SK-N-AS-TR and SH-EP-TR) were electroporated with pT-Rex-DEST30-YAP1^S127A^-2x/3xFlag or empty control vector (pT-Rex-pDest30). G418 was used to select transfected clones (600 µg/mL, SK-N-AS-TR-YAP1^S127A^; 800 µg/mL, SH-EP-TR-YAP1^S127A^) 72 h after electroporation. Enforced *YAP1^S127A^* expression was induced in the pcDNATM6/TR and pT-Rex-DEST30-YAP1^S127A^-2xFlag/3xFlag double-transfected cells by 4 μg/mL tetracycline (Sigma Aldrich, St. Louis, MO, USA) in the culture medium. Controls were treated with an equal volume of solvent (ethanol) in full medium (the highest ethanol concentration in any transfection was 0.1%). An empty vector control was used to account for possible YAP1-independent effects of tetracycline treatment on neuroblastoma cells. To reduce heterogeneity after transfection, single clones from all models were selected using fluorescence-activated cell sorting. Unless otherwise stated, data shown for the inducible YAP1^S127A^ models correspond to clone #10 for SH-EP-TR-YAP1^S127A^ and clone #2 for SK-N-AS-TR-YAP1^S127A^.

### 2.3. Assessing YAP1 Expression

Gene expression levels were determined by quantitative real-time polymerase chain reaction (qPCR) in 19 neuroblastoma cell lines. RNA was extracted using the RNeasy Mini Kit (Qiagen, Hilden, Germany) and processed using the Transcriptor First Strand cDNA Synthesis Kit (Roche, Basel, Switzerland) according to manufacturers’ protocols. The SYBR-green master mix kit (Roche) and gene-specific forward and reverse primers were used to perform qPCR in a StepOnePlus Real-Time PCR System (Applied Biosystems, Waltham, MA, USA). The expression levels were normalized to *SDHA* expression, known to be a reliable housekeeping gene in neuroblastoma cells [28]. Expression of endogenous *YAP1* in human cell lines was calculated as 2^−ΔCt^. Differential gene expression was determined as 2^−ΔΔCt^ for treated and control cells [29]. All samples were measured in technical duplicates. Experiments were performed as independent biological triplicates unless otherwise indicated. YAP1 protein levels were assessed by western blotting in 9 neuroblastoma cell lines and 4 additional cell line control models using ACTB or GAPDH as a loading control. Protein lysates were obtained by resuspending samples in lysis buffer (15 mM HEPES, 150 mM NaCl, 10 mM EGTA, 2% Triton X-100) and incubating on ice in an orbital shaker. Cell debris was removed via centrifugation, and the protein concentration in the supernatant was determined using the Pierce BCA Assay Kit (Thermo Fisher Scientific), following the manufacturer’s instructions. In total, 10–20 µg protein was separated on 10% SDS-PAGE gels and transferred to PVDF membranes (Hoffmann-La Roche). Immunoblots were blocked for 1 h in 5% milk powder in Tris-buffered saline and Tween 20 at 4 °C before incubating with antibodies (12–24 h, 4 °C) against YAP1 (1:5000, EP1674Y, Abcam, Cambridge, UK), ACTB (1:1000, sc-47778, Santa Cruz, Dallas, TX, USA) or GAPDH (1:5000, sc-25778, Santa Cruz). HRP-conjugated anti-rabbit IgG (for YAP1 and GAPDH, 1:5000; Dianova, Hamburg, Germany) or anti-mouse IgG (for ACTB, 1:5000; GE Dianova) secondary antibodies were incubated for 1 h at room temperature. Proteins were visualized using Amersham ECL Plus™ western blotting detection reagents (GE Healthcare, Chicago, IL, USA) and a UVchem Detection Device (Biometra, Jena, Germany). Band density was analyzed using ImageJ 1.47p software (Wayne Rasband, National Institute of Health, Bethesda, MD, USA) on western blots, and results were normalized to the respective loading controls.

### 2.4. Viability Assay

ATP quantification was performed in YAP1-inducible cell models using the CellTiter-Glo^®^ luminescent cell viability assay (Promega, Madison, WI, USA) in a 96-well format according to the manufacturer’s instructions. Per well, 10^4^ cells were seeded before inducing YAP1 expression for 72 h. Cultures were treated for 48 h with the chemotherapeutic agents: etoposide (0.65 nM to 50 µM for SH-EP cells, 0.1–150 µM for SK-N-AS cells), doxorubicin (0.65 nM to 10 µM for SH-EP cells, 10–50 µM for SK-N-AS cells), or vincristine (1 nM to 0.2 µM for SH-EP cells, 13 nM to 150 µM for SK-N-AS cells).

### 2.5. RNA Sequencing and Bioinformatic Analysis

Single neuroblastoma cell clones of tetracycline- or ethanol-treated SK-N-AS-TR-YAP1^S127A^ and SH-EP-TR-YAP1^S127A^ were subjected to RNA sequencing using an Illumina NextSeq 500 sequencer (Illumina, San Diego, CA, USA). Prior to library preparation, robust tetracycline-induced *YAP1*^S127A^ expression was confirmed by qPCR and western blot analysis in all samples. The mRNA library of three biological replicates per condition was prepared according to the Illumina TruSeq stranded mRNA protocol (Illumina). RNA sequencing was performed by the Charité/BIH Core Facility Genomics. RNA-sequencing data were aligned to the human GRCh38 genome assembly with GENCODE v27 annotation using the STAR aligner (version 2.5.4b) [30]. The number of reads per gene was determined using the FeatureCounts tool (version 1.6.1) [31] and normalized to the library size. Significantly differentially expressed genes were defined as those with an absolute fold-change mRNA abundance of ≥1 (Benjamini-Hochberg corrected *p*-value < 0.05) in YAP1-enhanced compared to control cells using the DESeq2 package (version 1.40.2) [32]. Volcano plots were generated using the EnhancedVolcano package (version 1.18.0) for R with a cut-off |log_2_ fold change| ≥ 1 with adjusted *p*-value < 0.05 [33]. Scatterplots were generated to visualize the log2 fold-change of differentially expressed genes in the two cell models in a Cartesian coordinate system. Genes significantly expressed in one of the two cell models were labeled by the name of the respective cell model. Genes significantly differentially expressed in both cell models were labeled concordant. Genes upregulated in one and downregulated in the other cell model were labeled discordant. A heatmap visualizing the Z-score of the top 50 significantly and concordantly differentially expressed genes was generated using the complex heatmap package for R (version 2.16.0.). Hierarchical clustering was applied to both genes and samples [34]. A gene ontology (GO) enrichment analysis for biological processes was performed on concordantly and significantly up- and downregulated genes in YAP1-induced SH-EP-TR-YAP1^S127A^ and SK-N-AS-TR-YAP1^S127A^ cells compared to controls using the clusterProfiler package for R [35]. The background was defined as genes with an average read count of at least 10 reads over all samples, excluding genes without any reads in any sample. A cnetplot visualizing the genes contributing to the top 5 GO terms was generated using the enrichplot software package (version 1.20.0) for R [36]. Single gene counts were plotted using the plotGenes function of the DESeq2 package [32]. Gene counts were normalized to fragments/kb/million mapped fragments (FPKM) in all samples, and the average from the three replicates was visualized in a scatterplot with log10 transformation. Genes were labeled mesenchymal (MES) or adrenergic (ADRN) according to the gene list provided by van Groningen et al. [19]. Genes assigned to neither the MES nor ADRN list were labeled neutral. Log2 fold changes of mesenchymal, adrenergic, and neutral genes were visualized in density and boxplot diagrams using ggplot2 [37]. To assess how YAP1 influences gene expression in a cohort of patients with neuroblastoma, we reanalyzed a previously published clinically annotated transcriptome dataset from 649 primary neuroblastomas (GSE45547) using the R2 Genomics Analysis and Visualization platform [38]. Analysis of gene expression positively correlated to YAP1 expression was conducted on all samples with an R cutoff > 0.4 and a Bonferroni corrected *p*-value < 0.05. To characterize the impact of MES gene expression on event-free survival, Cox proportional hazard ratios were calculated for MES genes.

### 2.6. Statistical Analysis

The median log2 fold changes of the MES, ADRN, and neutral gene subsets were compared using the Mann–Whitney U test [39], after determining a non-normal distribution using the Kolmogorov–Smirnov test [40]. To compare the number of concordantly regulated genes in both cell models with the number of discordantly regulated genes, a chi-squared test was performed [41]. Significant differences in metabolic activity were confirmed using the Wilcoxon matched-pairs signed rank test [42]. The association of high *YAP1* expression with event-free survival of patients with neuroblastoma was analyzed in the GSE45547 expression dataset from 649 primary neuroblastomas [43]. Kaplan–Meier diagrams comparing high and low *YAP1* expression were generated using the R2 Genomics Analysis and Visualization platform [38]. High *YAP1* expression was defined as expression in the upper quartile. Kaplan–Meier survival curves were generated using the R2 Genomics Analysis and Visualization platform, which performs log-rank (Mantel–Cox) tests for comparison of survival curves [44]. Chi-squared tests were performed to test for statistical significance. To assess whether YAP1-influenced gene expression in the primary neuroblastoma cohort (GSE45547 dataset) [43] was comparable to expression data generated from the cell models, we tested for enrichment of the respective significant gene sets using the hypergeometric test [45]. Whether enriched genes impacted event-free survival in patient data annotated in the GSE45547 dataset was tested using Cox proportional hazard ratios (R2 Genomics Analysis and Visualization platform [38]).

## 3. Results

### 3.1. Elevated YAP1 Expression in Neuroblastoma Correlates with Unfavorable Event-Free Survival

**Figure 1:**
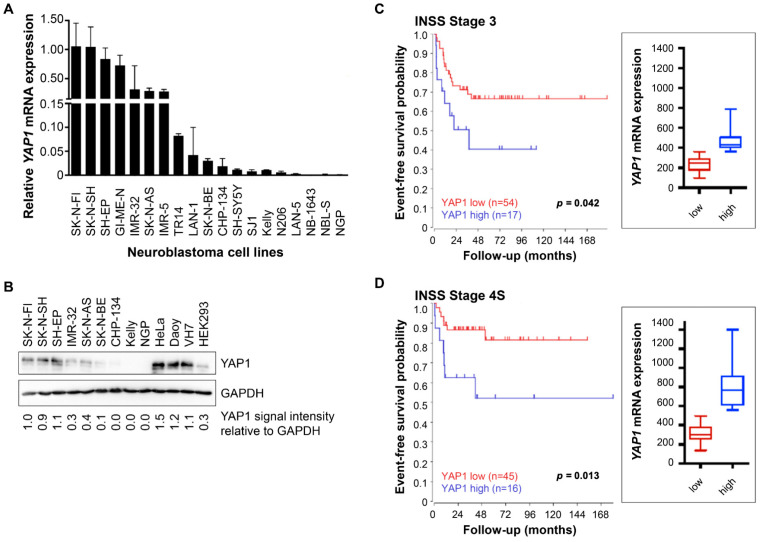
YAP1 expression varies among neuroblastoma cell lines, and high-level *YAP1* tumor expression correlates with unfavorable event-free survival in patients with INSS stage 3 and 4S disease. (**A**) *YAP1* mRNA expression was determined in a panel of 19 neuroblastoma cell lines using qPCR (mean ± standard deviation; n = 3). (**B**) YAP1 protein expression was determined by western blotting in nine neuroblastoma cell lines and four control cell lines. GAPDH served as loading control. (**C**,**D**) Kaplan–Meier diagrams demonstrating a correlation between high-level *YAP1* expression and unfavorable event-free survival of patients with neuroblastoma INSS stages 3 (**C**) and 4S (**D**). No significant associations were observed in other INSS stages. Diagrams were generated using the R2: Genomics Analysis and Visualization Platform. Statistical significance was assessed using the chi-squared test. Clinical and mRNA expression data were obtained from previously published data (GSE45547; n = 649 patients, Kocak et al., 2013) [43]. The uncropped Western blotting images can be found in Supplementary File S1.

Since YAP1 signaling has previously been associated with neuroblastoma relapse [6], we set out to assess the impact of YAP1 expression levels in primary neuroblastoma samples and cell lines. YAP1 mRNA and protein expression varied markedly across the 19 neuroblastoma cell lines assessed with qPCR (Figure 1A) and the 9 neuroblastoma cell lines assessed by western blotting (Figure 1B). YAP1 levels were highest in the SK-N-FI and SK-N-SH cell lines, and YAP1 was not expressed in the CHP-134, Kelly, and NGP cell lines (Figure 1A,B). *YAP1* expression did not correlate with the presence of *MYCN* amplification, *ALK* mutation, *TERT* rearrangement, or *ATRX* mutation in the cell lines. We reanalyzed the clinically annotated, previously published transcriptome dataset (GSE45547) [43] from samples collected at diagnosis of 649 primary neuroblastomas for correlations between *YAP1* expression in the tumor and patient survival. Stage-specific upper-quartile stratification separated cases to particularly group those with high *YAP1* tumor expression using cut points (INSS stages 1 and 2: 394.2, stage 3: 399.9, stage 4: 405.5, and stage 4S: 499.2). Elevated *YAP1* expression in tumor samples (upper quartile) significantly correlated with reduced event-free survival in these patients (INSS stage 3, *p* = 0.042, INSS stage 4S, *p* = 0.013, Figure 1C,D). No significant association between *YAP1* expression and event-free survival was observed in patients with INSS stage 1–2 or stage 4 disease (Supplementary Figure S1). To further quantify the association between *YAP1* expression and event-free survival, we performed Cox proportional hazards analyses for patients with INSS stage 3 and 4S neuroblastoma, which confirmed an increased risk of adverse events associated with high *YAP1* tumor expression (Supplementary Figure S2). To account for established clinical risk factors, we further stratified event-free survival analyses by *MYCN* status and age at diagnosis within INSS stage 3 and 4S cohorts, demonstrating that the association between high *YAP1* expression and reduced event-free survival persisted across these clinically relevant subgroups (Supplementary Figures S3 and S4).

Together, these data demonstrate marked variability in *YAP1* expression across neuroblastoma cell lines and indicate that high tumor *YAP1* expression is clinically relevant, correlating with unfavorable event-free survival in defined patient subgroups.

### 3.2. Enforcing YAP1 Activity Alters the Neuroblastoma Transcriptomic Landscape

**Figure 2:**
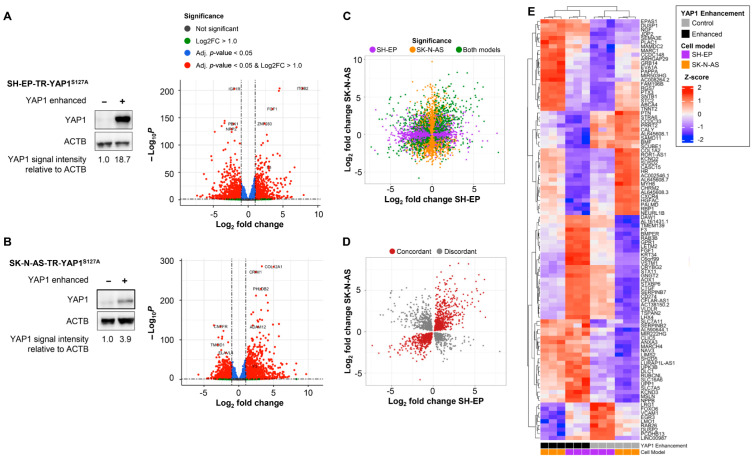
*YAP1* induction alters the neuroblastoma transcriptome. Western blot analysis confirmed increased YAP1 protein levels upon tetracycline-induced YAP1 enhancement in the two neuroblastoma cell models, SH-EP-TR-YAP1^S127A^ (**A**) and SK-N-AS-TR-YAP1^S127A^ (**B**). Volcano plots indicate Log2-fold changes and adjusted *p*-values for differentially expressed genes in YAP1-enhanced versus non-enhanced samples. Volcano plots were generated using the *Enhanced Volcano* package for R [33]. (**C**) Scatterplot comparing genes expressed in SK-N-AS-TR-YAP1^S127A^ against SH-EP-TR-YAP1^S127A^ Log2 fold changes. Genes with a *p*-value < 0.05 in either or both cell models were plotted. (**D**) Scatterplot comparing genes expressed in SK-N-AS-TR-YAP1^S127A^ against SH-EP-TR-YAP1^S127A^ Log2 fold changes. Genes significantly differentially expressed in both cell models are shown. The number of concordantly regulated genes was significantly higher than the number of discordantly regulated genes (chi-squared test, adjusted *p*-value). (**E**) Heatmap showing the Z scores of the top 50 differentially expressed and concordantly regulated genes in the YAP1-induced cell models compared to non-induced controls. The uncropped Western blotting images can be found in Supplementary File S1.

With YAP1 being a highly conserved transcription factor implicated in several essential cellular functions [7,8,9], we first assessed how enforced YAP1 activity affects the neuroblastoma transcriptome to better understand the particular functions affected by high YAP1 tumor levels. Based on established transcriptional and super-enhancer-based neuroblastoma cell-state classifications, SH-EP cells maintain a MES-like state, whereas SK-N-AS cells maintain an ADRN-like state [18,19]. Using this framework, a constitutively activated YAP1^S127A^ expression construct was employed to generate neuroblastoma cell models from established SH-EP and SK-N-AS cell lines. Expression was under the control of a tetracycline-inducible promoter in the cell models, enabling comparison with normal YAP1 levels in the cellular backgrounds of both ADRN and MES plastic states. RNA sequencing (Illumina NextSeq 500) was performed on single clones with and without enforced YAP1 activity to analyze changes in the neuroblastoma transcriptome enacted by enhanced YAP1 activity. Principal component analysis separated the two cell models at the first principal component, and models with enhanced YAP1 activity from the non-induced models at the second principal component (Supplementary Figure S5). Enforcing YAP1 activity significantly upregulated 707 genes and significantly downregulated 1130 genes in the SH-EP MES background (Figure 2A, Supplementary Table S1).

**Figure 3:**
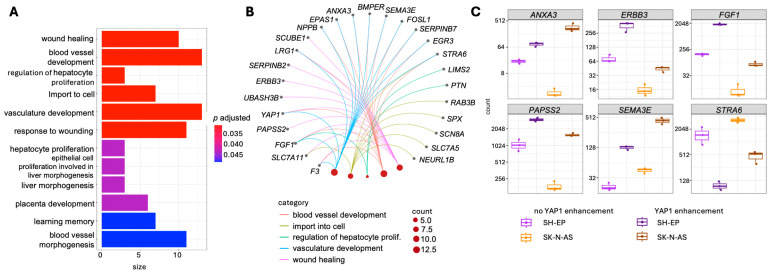
High-level *YAP1* expression alters gene expression patterns in neuroblastoma cells. (**A**) Gene ontology analysis for significantly differentially expressed and concordantly regulated genes in both neuroblastoma cell models. (**B**) CNET plot indicates the genes contributing for the top 5 significant Gene Ontology (GO) terms. The plot was generated using the *enrichplot* package for R [36]. (**C**) Boxplot diagrams of normalized gene counts for the genes of interest contributing to multiple GO terms. Normalization was performed using the *plotCounts* function of the *DESeq2* package for R.

In the SK-N-AS ADRN background, 1163 genes were significantly upregulated and 999 downregulated (Figure 2B, Supplementary Table S2). Genes regulated in the same direction upon YAP1 activation in both cell models were defined as concordantly regulated. Across all expressed genes, concordant regulation (n = 5987) significantly exceeded discordant regulation (n = 4927; Chi-squared test: X-squared = 102.95, df = 1, *p* < 2.2 × 10^−16^, Figure 2C). We repeated this analysis exclusively using genes that, singly, had significant expression changes (|log_2_ fold change| ≥ 1 with adjusted *p* < 0.05) upon YAP1 enhancement in both cell models. The number of concordantly regulated genes (n = 357) remained significantly higher than discordantly regulated (n = 170) genes (Chi-squared test: X-squared = 66.355, df = 1, *p* = 3.766 × 10^−16^; Figure 2D). Cell models with enforced YAP1 activity clustered together in unsupervised clustering of the Z-scores of concordantly regulated genes, emphasizing that YAP1 enhancement induces similar transcriptomic changes in both cell models (heatmap, Figure 2E). Gene ontology (GO) term analysis of concordantly up- and downregulated genes in both YAP1-enhanced cell models (compared to controls) revealed that wound healing and angiogenesis, among others, were significantly enriched biological processes (Figure 3A). Genes previously reported to be associated with cancer contribute to several GO terms and included *ANXA3*, *ERBB3*, *FGF1*, *PAPSS2*, *SMA3E* and *STRA6*. Interestingly, the upregulated *PAPSS2* and the *ANXA3* paralogs, *ANXA1*, *ANXA2*, *ANXA5* and *ANXA6*, previously reported to be associated with cancer, contribute to the MES score (Figure 3B,C) [19]. *PAPSS2* and *ANXA3* have been associated with breast cancer metastasis and migration [46,47], and other annexin genes have been associated with progression in several types of cancer [48]. We demonstrate that enforcing YAP1 activity strongly impacts the neuroblastoma transcriptome, predominantly by concordantly regulating gene expression in both the ADRN and MES cellular backgrounds.

### 3.3. Enforcing YAP1 Activity Induces a Mesenchymal Signature in Neuroblastoma Cell Models

**Figure 4:**
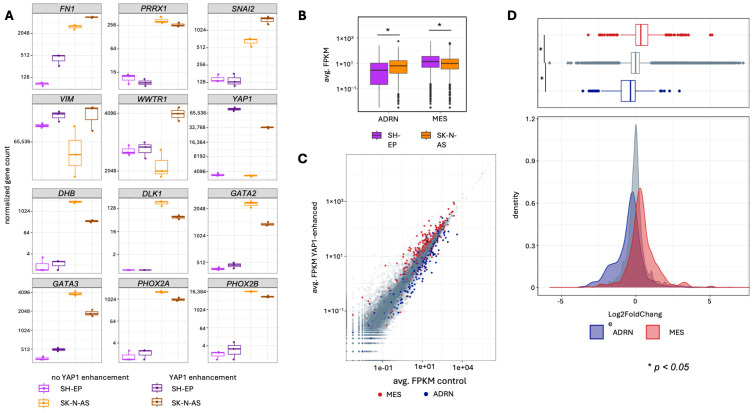
YAP1 induction promotes the expression of MES-associated genes in neuroblastoma cells. (**A**) Boxplot diagrams of normalized counts of genes associated with the MES and ADRN gene signature in neuroblastoma in the two neuroblastoma cell models [19]. (**B**) Boxplot diagrams mapping the expression of MES and ADRN gene sets in non-induced cell models. (**C**) Scatterplot of enhanced vs. non-enhanced FPKM-normalized gene counts in SK-N-AS-TR-YAP1^S127A^. (**D**) Boxplot and density plot mapping the Log2 fold change of neutral, MES, and ADRN gene subsets in SK-N-AS-TR-YAP1^S127A^. The median log2 fold change of gene subsets was compared using the Mann-Whitney U test.

Phenotypic plasticity towards a MES cell state has been reported to enable resistance to chemotherapy [19,20] and targeted drugs [21] in neuroblastoma cell models and may enable drug resistance in patients. We investigated whether YAP1, itself a key mesenchymal marker [49,50,51], could induce the MES gene signature and reduce chemosensitivity in our two inducible neuroblastoma cell models for enhanced YAP1 activity. We investigated MES signature expression (van Groningen and colleagues [19]) in RNA sequencing data from both SH-EP and SK-N-AS cell backgrounds. Normalized gene counts for the core MES markers were visualized for YAP1-enhanced and un-enhanced conditions in both cell backgrounds. Enhancing YAP1 activity in the SK-N-AS ADRN background markedly increased *FN1*, *SNAI2*, *VIM* and *WWTR1* expression (Figure 4A). Enhancing YAP1 activity in the SH-EP MES background increased *FN1* and *VIM* expression, while *SNAI2* and *WWTR1* expression remained unchanged (Figure 4A). YAP1^S127A^ expression slightly diminished *PRRX1* expression, a gene that joins *YAP1* as part of the gene regulatory circuit for mesenchymal reprogramming in both cell models (Figure 4A). Expression of the core ADRN markers (*DBH*, *DLK1*, *GATA2*, *GATA3*, *PHOX2A* and *PHOX2B*) was reduced in the YAP1-enhanced SK-N-AS ADRN background, and expression remained unchanged in the YAP1-enhanced SH-EP MES cell background (Figure 4A).

To assess baseline expression of core genes associated with the MES and ADRN cell states [19], log-transformed fragment/kb/million mapped fragment (FPKM) values from the control condition were plotted. As expected for the SH-EP MES background, MES-associated gene expression was significantly higher compared to ADRN-associated gene expression (Wilcoxon rank sum test, W = 127,877, *p* < 2.2 × 10^−16^) in uninduced SH-EP cells (Figure 4B). When we compared gene expression in the two uninduced cell backgrounds, expression of MES-associated genes was significantly higher (Wilcoxon rank sum test, W = 128,095, *p* = 0.003964) and the expression of ADRN-associated genes significantly lower (Wilcoxon rank sum test, W = 46,216, *p* = 6.756 × 10^−12^) in SHEP compared to SK-N-AS cells (Figure 4B). ADRN-associated genes were expressed at higher levels in SK-N-AS cell models, consistent with an adrenergic-like baseline state. Our baseline transcriptomic data confirm that SH-EP cells maintain a MES-type identity, whereas SK-N-AS cells maintain an ADRN-type identity under untreated control conditions, in line with previously published neuroblastoma cell-state profiles [19]. Gene expression between the control and YAP-enhanced conditions was stably correlated based on plotted FPKM replicate averages (Figure 4C).

**Figure 5:**
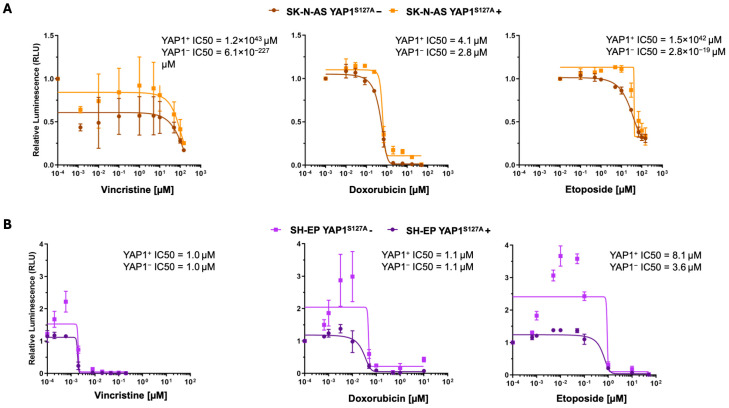
Enhanced *YAP1* expression in an adrenergic background renders neuroblastoma cells less sensitive to chemotherapy. Boxplot diagrams indicate the fold change in cell viability (treated versus untreated) measured by the CellTiter-Glo assay in YAP1-enhanced and non-enhanced conditions. Treatment was performed singly using the chemotherapeutic agents etoposide, doxorubicin, and vincristine employing the SH-EP-TR-YAP1^S127A^ (**A**) and SK-N-AS-TR-YAP1^S127A^ (**B**) cell models.

Collectively, YAP1 activation shifted gene expression toward a mesenchymal program, with increased MES-associated and reduced ADRN-associated gene expression (compared to average expression, respectively). When we compared log2 fold changes of the respective gene sets in the SK-N-AS ADRN background, enhancing YAP1 significantly increased MES gene set expression compared to neutral (Wilcoxon rank sum test: W = 3,093,111, *p* < 2.2 × 10^−16^) or ADRN gene sets (Wilcoxon rank sum test: W = 130,303, *p* < 2.2 × 10^−16^). The ADRN gene set was significantly less expressed (Wilcoxon rank sum test: W = 4,783,557, *p* < 2.2 × 10^−16^) than the neutral gene set (Figure 4D). MES and ADRN gene expression in the SH-EP background remained unchanged upon enforcing YAP1 activity (Supplementary Figure S6). To functionally confirm MES features in YAP1-enhanced cell models, we tested cell viability after treating the cell models with the chemotherapeutic agents etoposide, doxorubicin, or vincristine (singly). Enhancing YAP1 rendered cells less sensitive to all three chemotherapy drugs in the SK-N-AS ADRN cell background, which is known to exhibit comparatively low baseline chemosensitivity among neuroblastoma models ([52], Figure 5A,B). In the SH-EP cell background, which is already maintained in the MES state, chemotherapy-induced reductions in viability were observed, but inducing *YAP1* expression did not further augment resistance beyond this baseline MES-associated phenotype (Figure 5A,B). Our findings demonstrate that enhancing YAP1 activity significantly increases key MES marker and MES signature expression, as well as enhances resilience to clinically used chemotherapy agents in an ADRN neuroblastoma cell background.

### 3.4. YAP1-Associated Changes in the Neuroblastoma Cell Models Significantly Overlap with Genes Whose Expression Correlates with YAP1 in Tumors

**Figure 6:**
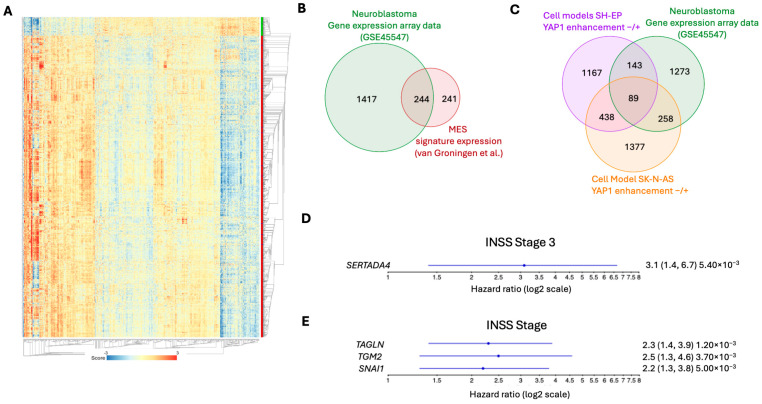
*YAP1* downstream target gene expression correlates with the MES gene signature in neuroblastoma tumors. (**A**) Heatmap of genes significantly correlated with *YAP1* expression in tumor data [43]. Venn diagrams illustrating the overlap between *YAP1*-correlated genes in GSE45547 and MES genes [19]. (**B**) Hypergeometric test, *p* = 1.961398 × 10^−122^) and *YAP1*-correlated genes in GSE45547 and both cell models. (**C**) Hypergeometric test, SK-N-AS *p* = 1.50965 × 10^−25^; SH-EP *p* = 8.797828 × 10^−5^. Cox proportional hazard analysis of YAP1-correlated genes significantly correlated with reduced event-free survival for patients with INSS Stage 3 (**D**) and 4s tumors (**E**). Hazard ratios are displayed with a 95% confidence interval and *p*-value.

To evaluate whether gene expression changes induced by YAP1 activity in our cell models are representative of gene expression in primary neuroblastomas, we compared our in vitro results to the expression array data (GSE45547) from the previously analyzed 649 primary neuroblastomas. Of the 17,654 genes expressed in these primary neuroblastoma samples [50], 1763 genes correlated significantly with *YAP1* expression (1661 genes upregulated, 102 genes downregulated; Figure 6A, Supplementary Table S3). Of all 485 genes included in the MES signature score by van Groningen et al. [19], 244 were significantly positively correlated to *YAP1* expression in the GSE45547 expression dataset, demonstrating a highly significant enrichment (Figure 6B; Hypergeometric test, *p* = 1.961398 × 10^−122^). Differentially expressed genes in both cell models significantly overlapped with the list of significantly *YAP1*-correlated genes in the primary neuroblastoma samples (GSE45547). The overlap between YAP1-regulated genes in SK-N-AS-TR-YAP1^S127A^ and the GSE455447 dataset was 347 (Figure 6C, Hypergeometric test, *p* = 1.50965 × 10^−25^) and 232 in SH-EP-TR-YAP1^S127A^ and GSE455447 (Figure 6C, Hypergeometric test, *p* = 8.797828 × 10^−5^). The overlap between all three datasets was 89 genes, indicating shared YAP1-dependent gene regulation across cell models and primary tumors (Figure 6C, Supplementary Table S4). Of these 89 genes, high *SERTAD4* expression significantly correlated with reduced event-free survival in patients with INSS stage 3 neuroblastomas (Figure 6D, Cox proportional hazard ratio, HR = 3.1, *p* = 0.0054). High expression of *TAGLN* (Cox proportional hazard ratio, HR = 2.3, *p* = 0.0012), *TMG2* (Cox proportional hazard ratio, HR = 2.5, *p* = 0.0037), and *SNAI1* (Cox proportional hazard ratio, HR = 2.2, *p* = 0.005) was significantly correlated with reduced event-free survival in patients with INSS stage 4s neuroblastomas (Figure 6E). The Cox proportional hazard ratio for unfavorable event-free survival in patients was significantly higher for 18 genes from the van Groningen MES gene signature [19] in INSS stage 3 neuroblastomas and for 39 genes in INSS stage 4S neuroblastomas, indicating that unfavorable event-free survival was associated with high MES-associated gene expression. Altogether, we demonstrate that the gene expression patterns displayed by the cell models generated in this study overlap with gene expression in a previously analyzed cohort of primary neuroblastomas. High expression of selected MES-associated genes correlated with unfavorable event-free survival in patients with INSS stages 3 and 4S disease.

## 4. Discussion

Here we demonstrate that *YAP1* expression enhances MES gene expression in neuroblastoma cells and significantly correlates with reduced event-free survival in patients with INSS stage 3 or 4S neuroblastoma. In our cell models, RNA sequencing revealed that YAP1^S127A^ induction altered the expression of genes associated with biological processes such as wound healing and vasculature development. Enforced YAP1 activation increased the expression of MES-associated genes. The set of MES genes and genes differentially expressed upon YAP1 induction significantly overlapped with genes whose expression correlated with *YAP1* expression in the GSE45547 patient dataset. These findings suggest that YAP1 may drive MES-like features in tumors and contribute to reduced event-free survival in specific neuroblastoma subgroups. Stratifying patients by tumor stage revealed that elevated *YAP1* expression was associated with significantly reduced event-free survival in INSS stages 3 and 4S. No significant association was observed for INSS stage 4 tumors. Most INSS stage 4 tumors are driven by potent oncoproteins (MYCN [53,54], TERT [53,55], ATRX [53,54], ALK [53,56], RAS/MAPK [53,54]), which may reduce the impact of YAP1 on survival. In contrast, the generally favorable prognosis of INSS stage 4S tumors can be attributed to spontaneous regression in these tumors [57]. High *YAP1* expression may interfere with processes mediating this regression. Additionally, YAP1’s role in impairing neural differentiation and other organs may promote an undifferentiated MES-like cell state [58,59], consistent with our model data. In line with this, YAP1 was shown to induce an undifferentiated neural crest-like phenotype in neuroblastoma cells [17]. By promoting an undifferentiated MES-like cell state, high YAP1 activity potentially contributes to hampering spontaneous regression.

We enforced high-level YAP1 activity in the two different plastic states neuroblastoma cells [18,19] can exist in using cell models produced from human cell lines known to maintain an ADRN state (SK-N-AS) and a MES state (SH-EP). Higher MES and lower ADRN gene expression was confirmed in the non-induced MES model. Enforced high-level YAP1 activity significantly increased MES gene expression in the non-MES background, but not in the MES background. The PRRX1 core regulatory circuitry induces a MES state in neuroblastoma cell lines [19]. In our data, YAP1 induction was followed by significantly increased MES gene expression, interestingly lacking differential expression of PRRX1. In line with our data, other studies have reported reduced MES gene expression after *YAP1* knockdown in neuroblastoma cell lines [51]. Taken together, these findings are compatible with YAP1 being a downstream effector of the PRRX1 core regulatory circuitry, able to induce MES-like gene expression patterns in neuroblastoma.

MES-like neuroblastoma cells have been reported to be resistant to traditional chemotherapy, immunotherapy, and targeted approaches [51,60,61]. A post-therapy increase in YAP1 has been proposed to be a reaction to therapy rather than the selection of YAP1-expressing cells [51]. Our data emphasize the potential of YAP1 to induce a mesenchymal-like state in neuroblastoma cells. The post-therapy increase in YAP1 expression might contribute to enriching MES-type cells and enhancing intra-tumor heterogeneity. While our short-term functional assays assessing cell viability support an association with reduced chemosensitivity, further studies using orthogonal readouts will be required to dissect the underlying mechanisms. Enhancing therapy resistance is one possible mechanism by which YAP1 activity could contribute to reduced event-free survival. We acknowledge that our study does not fully delineate the mechanisms of YAP1-associated drug response, representing a limitation and future direction. Beyond inducing a mesenchymal-like state, YAP1 may also influence chemotherapy sensitivity via apoptotic regulators (e.g., BCL2 family, Survivin) and DNA damage-related pathways. In line with this, exploratory apoptosis analyses in YAP1-modulated neuroblastoma cell models did not reveal a pronounced YAP1-dependent anti-apoptotic phenotype, indicating that altered drug response may instead reflect broader changes in cellular state or stress adaptation (Supplementary Figures S7 and S8).

To isolate, as best as possible, the transcriptomic mechanisms through which YAP1 activity wields its influence, we made use of single clones from the cell models (Supplementary Figures S9 and S10). Still, YAP1-associated effects are context dependent and likely shaped by the underlying cellular state, rather than being conserved across all neuroblastoma cell line models. We realize that results from cell models are not always reproducible in real-world data. TEAD inhibitors, which pharmacologically disrupt Hippo–YAP/TEAD signaling [62], or transcriptionally impaired YAP1 variants, which would have the same effect, could directly assess the mechanistic link between YAP1 activity and MES program activation. This approach is warranted to fully understand the mechanism of action. Several pharmacological strategies have been developed to target YAP1 signaling, ranging from direct inhibitors of YAP–TEAD interaction (e.g., verteporfin [63],or VT3989 [62]) to kinase inhibitors like dasatinib and pazopanib that impact YAP1/TAZ activity [64]. These compounds remain largely in preclinical or early clinical evaluation so far. However, we could show that there is a significant overlap between YAP1-regulated genes in our cell models and *YAP1*-associated genes in previously published gene expression data from patients, underlining the strong regulatory effects YAP1 activity has in both cell models and patients. Altogether, our study sheds further light on the transcriptomic mechanisms underlying the MES phenotype. The interaction of the previously reported transcriptional mechanisms driving transdifferentiation towards a MES-like state remains incompletely understood. Future studies could address this by enhancing and inhibiting MES transcription factors in several combinations to assess their interplay in regulating MES gene expression and phenotype.

## 5. Conclusions

Here we show a YAP1-dependent increase in MES gene expression that was replicated in real-world neuroblastoma gene expression data. In contributing to phenotypic plasticity, YAP1 might be an interesting target for future investigations of how resistance to cancer therapy is mediated.

## Data Availability

RNA-sequencing data from cell lines are available at the NCBI Sequence Read Archive (http://www.ncbi.nlm.nih.gov/bioproject/1172331, accessed on 24 September 2024). The RNA expression dataset from tumors used for reanalysis can be accessed via NCBI (https://www.ncbi.nlm.nih.gov/geo/query/acc.cgi?acc=GSE45547, accessed on 24 September 2024).

## Supplementary Materials

The following supporting information can be downloaded at https://www.mdpi.com/article/10.3390/cancers18030383/s1: Figure S1. Kaplan–Meier curves comparing YAP1 mRNA expression in neuroblastoma INSS stages 1 and 2 as well as stage 4 disease with event-free survival of patients. Figure S2. YAP1 expression is associated with adverse event-free survival in specific neuroblastoma subgroups. Cox proportional hazards models were used to estimate hazard ratios for the association between high YAP1 tumor expression and event-free survival in a cohort of primary neuroblastoma patients (GSE45547). Analyses were performed separately for patients with INSS stage 3 disease (top) and INSS stage 4S disease (bottom). High YAP1 expression was defined using the upper-quartile cut-point within each stage-specific cohort. Hazard ratios >1 indicate an increased risk of adverse events associated with elevated YAP1 expression. Figure S3: Event-free survival stratified by YAP1 expression in INSS stage 3 neuroblastoma according to MYCN status or age at diagnosis. Kaplan–Meier event-free survival (EFS) curves for patients with INSS stage 3 neuroblastoma stratified by tumor YAP1 expression (upper quartile) and MYCN status (top left, MYCN-amplified vs. top right, non-amplified) or age at diagnosis (bottom left, <18 months vs. bottom right, ≥18 months). Survival analyses were performed using the R2 Genomics Analysis and Visualization Platform based on the GSE45547 dataset. Statistical significance was assessed using the Mantel–Cox log-rank test. Figure S4: Event-free survival stratified by YAP1 expression in INSS stage 4S neuroblastoma according to MYCN status or age at diagnosis. Kaplan–Meier event-free survival (EFS) curves for patients with INSS stage 4S neuroblastoma stratified by tumor YAP1 expression (upper quartile) and MYCN status (left, MYCN-amplified vs. right, non-amplified). Assessment of INSS stage 4S together with <18 months vs. ≥18 months was not possible because stage 4S only occurs in patients diagnosed up to 18 months of age, by definition. Survival analyses were performed using the R2 Genomics Analysis and Visualization Platform based on the GSE45547 dataset. Statistical significance was assessed using the Mantel–Cox log-rank test. Figure S5. Principal component analysis projecting the cell model-derived RNA sequencing data onto two dimensions. Figure S6. Boxplot and density plots of log-2 fold changes of MES and ADRN gene expression in the SH-EP cell background indicates no significant changes in gene expression upon YAP1 enhancement in the SH-EP cell background. Figure S7. Apoptosis is only marginally affected by YAP1 siRNA knockdown. Establishing a siRNA-mediated YAP1 knockdown in neuroblastoma cells (left). YAP1 mRNA expression was analyzed by qPCR (displayed in relation to SDHA expression) after a transient YAP1 knockdown with two YAP1-targeting siRNAs (siYAP1 #1, #2) and two non-target control siRNAs (NC #1, #2), 72 h after siRNA transfection. Cell death ELISA in 4 neuroblastoma cell lines (middle). Flow cytometry analysis applying PI/Annexin V staining to SK-N-SH 72 h after siRNA transfection to assess apoptosis upon YAP1 knockdown (right). Bars represent mean ± SD of n = 3 independent experiments. Statistical significance was assessed using the Mann-Whitney U test; * 0.01 < *p* ≤ 0.05; ** 0.001 < *p* ≤ 0.01. Figure S8. YAP1S127A activation in neuroblastoma cells only had a minor affect on apoptosis. Shown are different SH-EP-TR-YAP1S127A and SK-N-AS-TR-YAP1S127A clones (#) and empty vector controls (LV) after tetracycline (+Tet) or solvent (ethanol, -Tet) treatment for 72 h. Flow cytometry analysis of YAP1-activated (+Tet) and ethanol-treated (-Tet) control cells for SH-EP-TR-YAP1S127A (left) and SK-N-AS-TR-YAP1S127A clones (right) assessing proportions of PI/Annexin V double-positive dead cells and Annexin V single-positive apoptotic cells. Bar plots represent mean ± SD of n = 3. Statistical significance was assessed using the Mann-Whitney U test. Figure S9. Cell viability of YAP1-activated cells under treatment with etoposide, doxorubicin and vincristine. Two YAP1-inducible cell lines were treated with different concentrations of etoposide, doxorubicin and vincristine. SH-EP-TR-YAP1S127A clone #10 viability was significantly higher under treatment of any single agent of the three when YAP1S127A was activated (+Tet) compared to solvent control (-Tet, blue top panel). No difference was detected for clone #14. Both SK-N-AS TR-YAP1S127A cell clones were more viable upon doxorubicin, etoposide or vincristine treatment, when YAP1S127A was overexpressed (yellow bottom panel). Bars represent mean enrichment over the untreated controls (±SD, n = 3). *P*-values were calculated using the Wilcoxon matched-pairs signed rank test. *p* *0.01 < *p* < 0.05, **0.001 < *p* < 0.01. Figure S10. Lost YAP1 activation in SH-EP-TR-YAP1S127A clone #14. YAP1 gene expression level in induced SH-EP-TR-YAP1S127A (left) and SK-N-AS-YAP1S127A (right) cell clones are shown after treatment and simultaneous harvest with chemotherapeutically treated cells. Quantitative PCR detected no YAP overexpression in comparison to the empty vector control (LV) in clone #14. Bars represent expression enrichment in YAP1-activated cells over solvent controls (means ± SD) in relation to SDHA expression as a control for equivalent cell numbers. Table S1. Genes significantly up- and downregulated in the SH-EP background. Table S2. Genes significantly up- and downregulated in the SK-N-AS background. Table S3. Overview of genes significantly correlating with YAP1 expression in the GSE45547 tumor cohort. Table S4. Overlap of significantly differentially expressed genes in both cell models and YAP1-correlated genes in the GSE45547 tumor cohort. File S1. The uncropped original Western blotting images.

## Abbreviations

The following abbreviations are used in this manuscript:

MES	mesenchymal
ADRN	adrenergic
TR	Tet-repressor
qPCR	real-time polymerase chain reaction
GO	gene ontology
FPKM	fragments/kb/million mapped fragments
