# Supplementary material for: YAP1 Enhances Mesenchymal-Type Gene Expression in Human Adrenergic-Type Neuroblastoma Cells
Source: Cancers (Basel). 2026 Jan 26;18(3):383. doi: 10.3390/cancers18030383 (PMC12897277; doi:10.3390/cancers18030383)
Supplement: Supplementary file 1 [file cancers-18-00383-s001.zip › Supplementary figures.pdf]

Supplementary Figures

YAP1 Enhances Mesenchymal-Type Gene Expression in Human Adrenergic-Type Neuroblastoma Cells

Marius Ludwig [1,2,†], Kerstin Ahrens [1,†], Annika Winkler [1], Jasmin Wünschel [1], Peris Ruka [1], Marco Lodrini [1], Falk Hertwig [1], Sveva Castelli [1], Theresa M. Thole-Kliesch [1], Jan F. Hollander [1,2], Steffen Fuchs [1,2,3], Annette Künkele [1,2,3], Marvin Jens [1], Soulafa Mamlouk [4], Steven W. Warmann [4], Kathy Astrahantseff [1], Angelika Eggert [1,2,3,5], Johannes H. Schulte [3,6], Annabell Szymansky [1,‡] and Hedwig E. Deubzer [1,2,3,7,*,‡]

[1] Department of Pediatric Oncology and Hematology, Charité-Universitätsmedizin Berlin, Corporate Member of Freie Universität Berlin and Humboldt-Universität zu Berlin, Campus Virchow Klinikum, 13353 Berlin, Germany; marius.ludwig@charite.de (M.L.); annika.winkler@dhzc-charite.de (A.W.); jasmin.wuenschel@charite.de (J.W.); peris.ruka@charite.de (P.R.); marco.lordini@charite.de (M.L.); sveva.castelli@charite.de (S.C.); theresa.thole-kliesch@charite.de (T.M.T.-K.); jan-fredrick.hollander@charite.de (J.F.H.); steffen.fuchs@charite.de (S.F.); annette.kuenkele@charite.de (A.K.); marvin.jens@charite.de (M.J.); kathy.astrahantseff@charite.de (K.A.);. annabell.szymansky@charite.de (A.S.)

[2] Berlin Institute of Health (BIH) at Charité, 10178 Berlin, Germany

[3] German Cancer Consortium (DKTK), Partner Sites Berlin and Tübingen, German Cancer Research Center (DKFZ), 69120 Heidelberg, Germany

[4] Department of Pediatric Surgery, Charité-Universitätsmedizin Berlin, Corporate Member of Freie Universität Berlin and Humboldt-Universität zu Berlin, Campus Virchow Klinikum, 13353 Berlin, Germany; soulafa.mamlouk@charite.de (S.M.); steven.warmann@charite.de (S.W.W.)

[5] University Hospital Essen, 45147 Essen, Germany, angelika.eggert@uk-essen.de

[6] Department of Pediatric Oncology and Hematology, University Hospital Tübingen, 72076 Tübingen, Germany; johannes.schulte@med.uni-tuebingen.de

[7] Experimental and Clinical Research Center (ECRC) of Charité and Max-Delbrück-Center of Molecular Medicine in the Helmholtz Association, Lindenberger Weg 80, 13125 Berlin, Germany

* Correspondence: hedwig.deubzer@charite.de

† These authors contributed equally to this work as co-first authors.

‡ These authors contributed equally to this work as co-senior authors.

Table of Contents

Supplementary Figure S1 p.2
Supplementary Figure S2 p.3
Supplementary Figure S3 p.4
Supplementary Figure S4 p.5
Supplementary Figure S5 p.6
Supplementary Figure S6 p.7
Supplementary Figure S7 p.8
Supplementary Figure S8 p.9
Supplementary Figure S9 p.10
Supplementary Figure S10 p.11

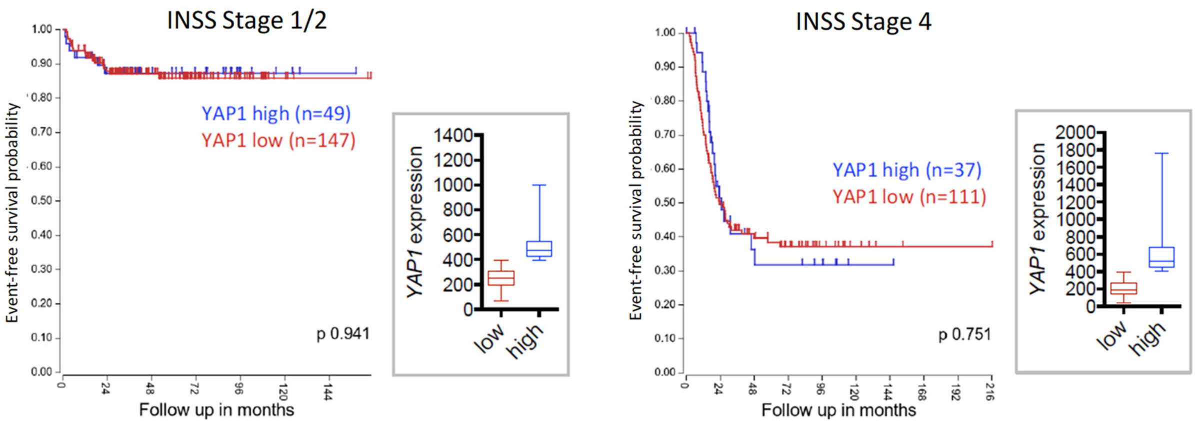

Supplementary Figure S1: Kaplan-Meyer curves comparing *YAP1* mRNA expression in neuroblastoma INSS stages 1 & 2 (**left**) as well as stage 4 disease (**right**) with event-free survival of patients showed no significant correlations.

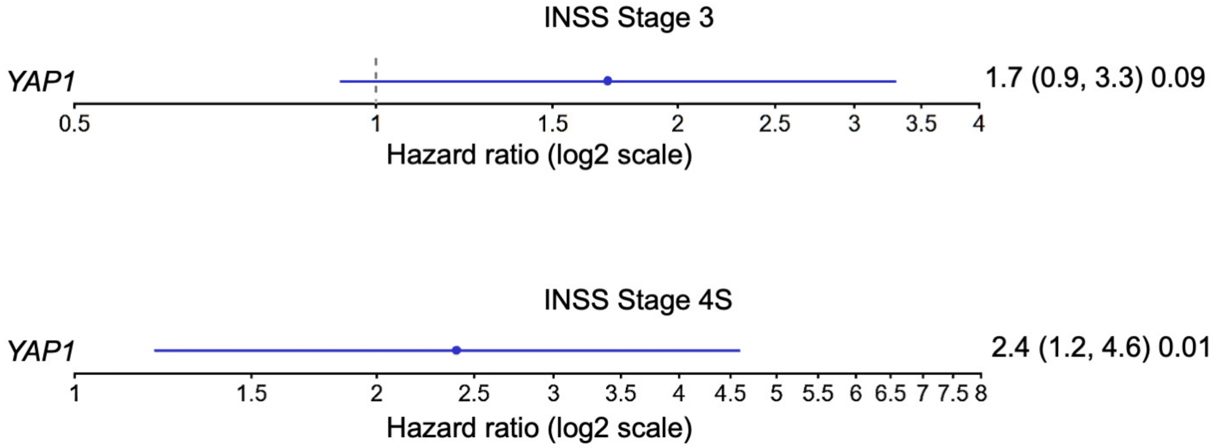

Supplementary Figure S2: *YAP1* expression is associated with adverse event-free survival in specific neuroblastoma subgroups. Cox proportional hazards models were used to estimate hazard ratios for the association between high *YAP1* tumor expression and event-free survival in a cohort of primary neuroblastoma patients (GSE45547). Analyses were performed separately for patients with INSS stage 3 disease (**top**) and INSS stage 4S disease (**bottom**). High *YAP1* expression was defined using the upper-quartile cut-point within each stage-specific cohort. Hazard ratios >1 indicate an increased risk of adverse events associated with elevated *YAP1* expression.

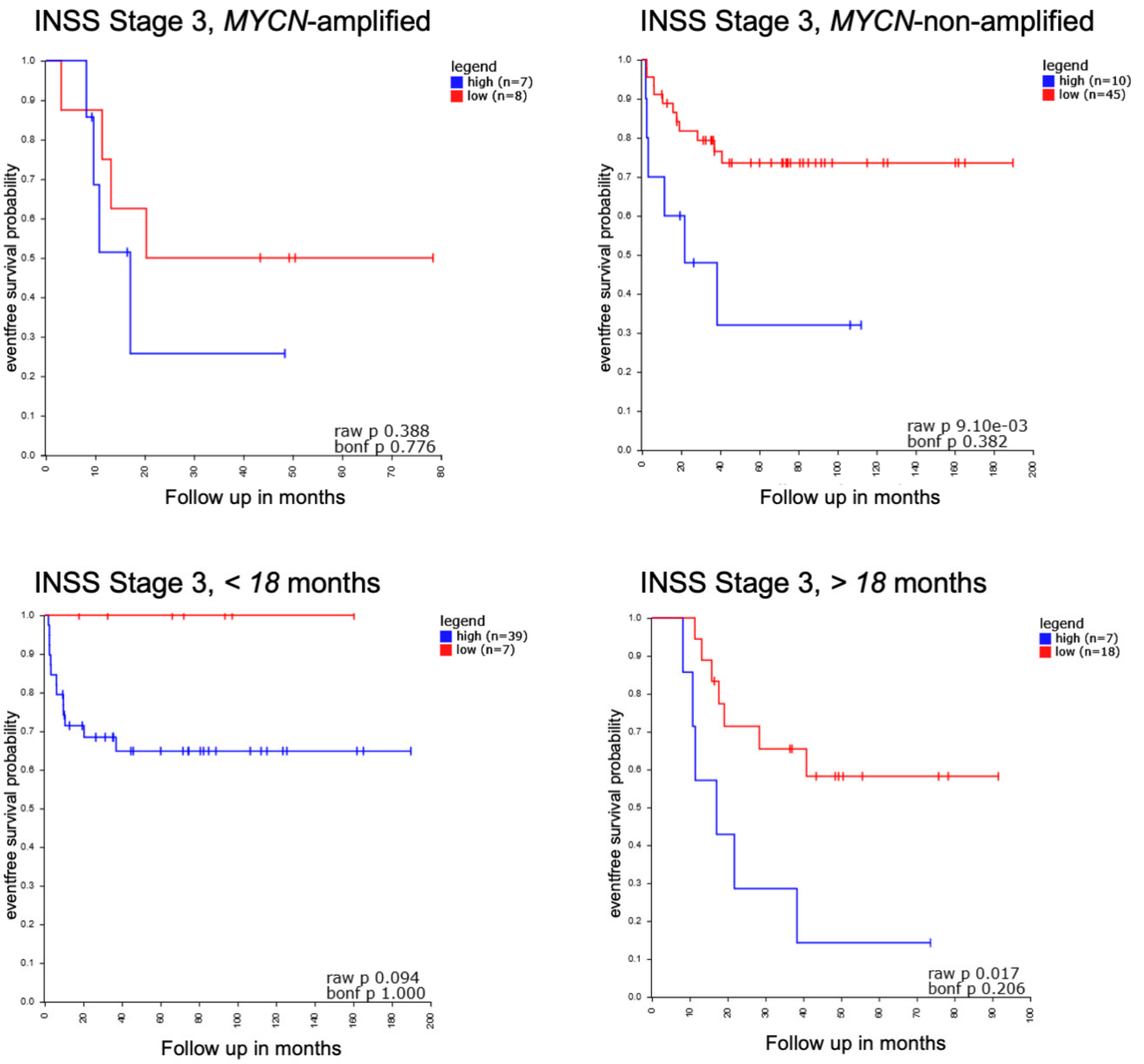

Supplementary Figure S3: Event-free survival stratified by *YAP1* expression in INSS stage 3 neuroblastoma according to MYCN status or age at diagnosis. Kaplan–Meier event-free survival (EFS) curves for patients with INSS stage 3 neuroblastoma stratified by tumor *YAP1* expression (upper quartile) and *MYCN* status (**top left**, *MYCN*-amplified vs. **top right**, non-amplified) or age at diagnosis (**bottom left**, <18 months vs. bottom right, ≥18 months). Survival analyses were performed using the R2 Genomics Analysis and Visualization Platform based on the GSE45547 dataset. Statistical significance was assessed using the Mantel–Cox log-rank test.

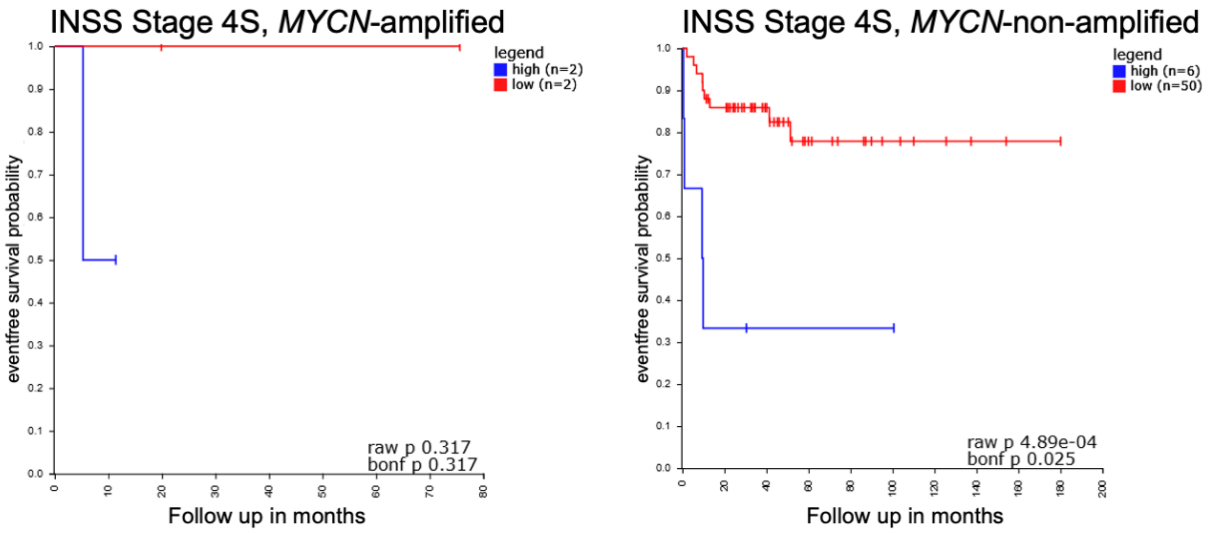

Supplementary Figure S4: Event-free survival stratified by *YAP1* expression in INSS stage 4S neuroblastoma according to *MYCN* status or age at diagnosis. Kaplan–Meier event-free survival (EFS) curves for patients with INSS stage 4S neuroblastoma stratified by tumor *YAP1* expression (upper quartile) and *MYCN* status (**left**, *MYCN*-amplified vs. **right**, non-amplified). Assessment of INSS stage 4S together with <18 months vs. ≥18 months was not possible because stage 4S only occurs in patients diagnosed up to 18 months of age, by definition. Survival analyses were performed using the R2 Genomics Analysis and Visualization Platform based on the GSE45547 dataset. Statistical significance was assessed using the Mantel–Cox log-rank test.

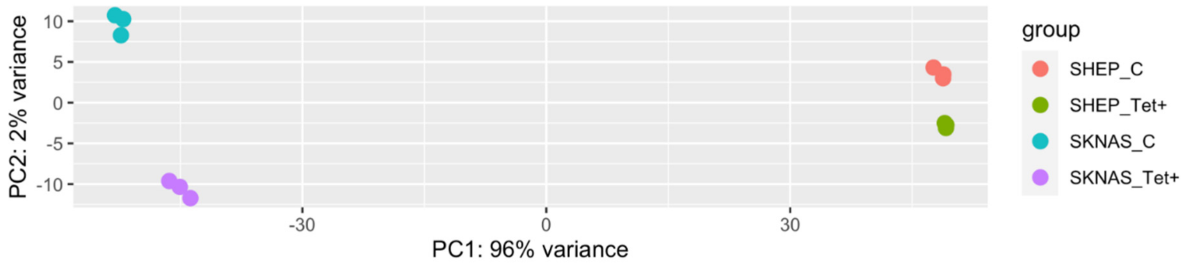

Supplementary Figure S5: Principal component analysis projecting the cell model-derived RNA sequencing data onto two dimensions. Samples were separated in the first principle component (PC) by cell line identity and into YAP1 enhanced and non-enhanced samples in the second principal component. C, control. Tet+, YAP1 enhancement by tetracycline addition to the cell culture medium.

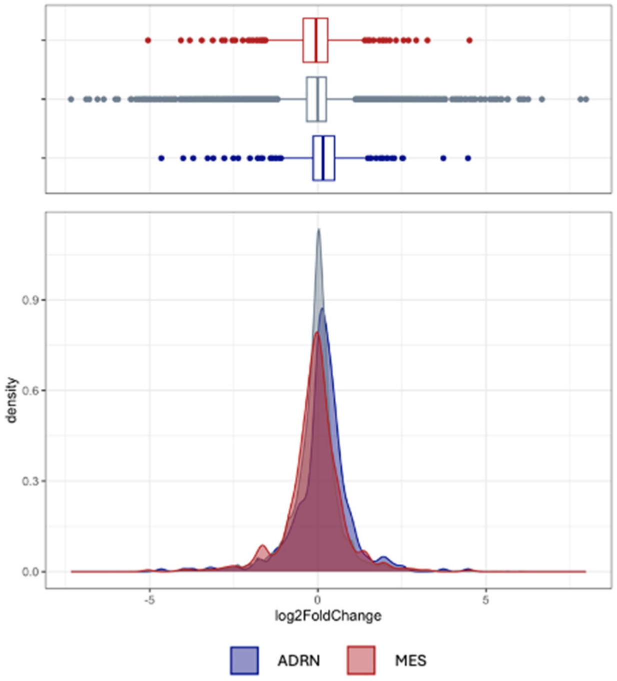

Supplementary Figure S6: Boxplot and density plots of log2-fold changes of MES and ADRN gene expression in the SH-EP cell background indicates no significant changes in gene expression upon YAP1 enhancement in the SH-EP cell background.

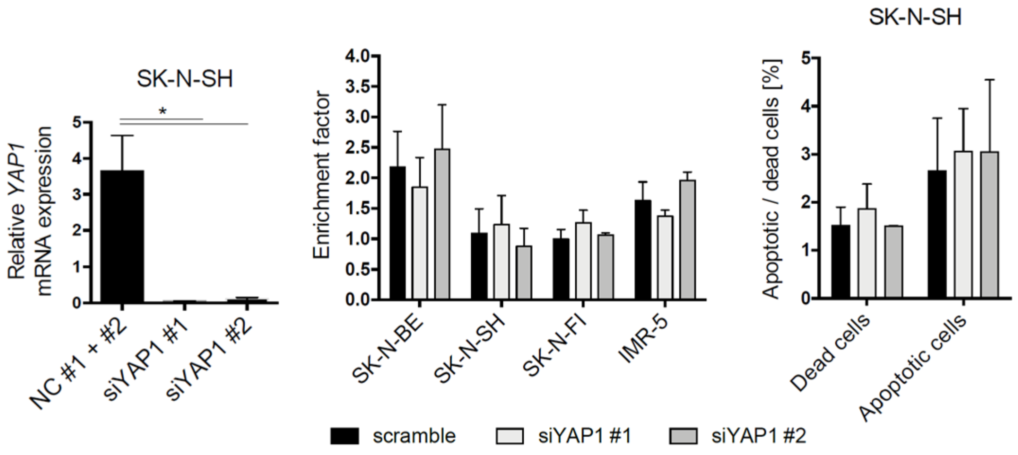

Supplementary Figure S7: Apoptosis is only marginally affected by *YAP1* siRNA knockdown. Establishing a siRNA-mediated *YAP1* knockdown in neuroblastoma cells (**left**). *YAP1* mRNA expression was analyzed by qPCR (displayed in relation to *SDHA* expression) after a transient *YAP1* knockdown with two YAP1-targeting siRNAs (siYAP1 #1, #2) and two non-target control siRNAs (NC #1, #2), 72 hours after siRNA transfection. Cell death ELISA in 4 neuroblastoma cell lines (**middle**). Flow cytometry analysis applying PI/Annexin V staining to SK-N-SH 72 hours after siRNA transfection to assess apoptosis upon YAP1 knockdown (**right**). Bars represent mean ±SD of n=3 independent experiments. Statistical significance was assessed using the Mann-Whitney U test; * 0.01 < p ≤ 0.05; ** 0.001 < p ≤ 0.01.

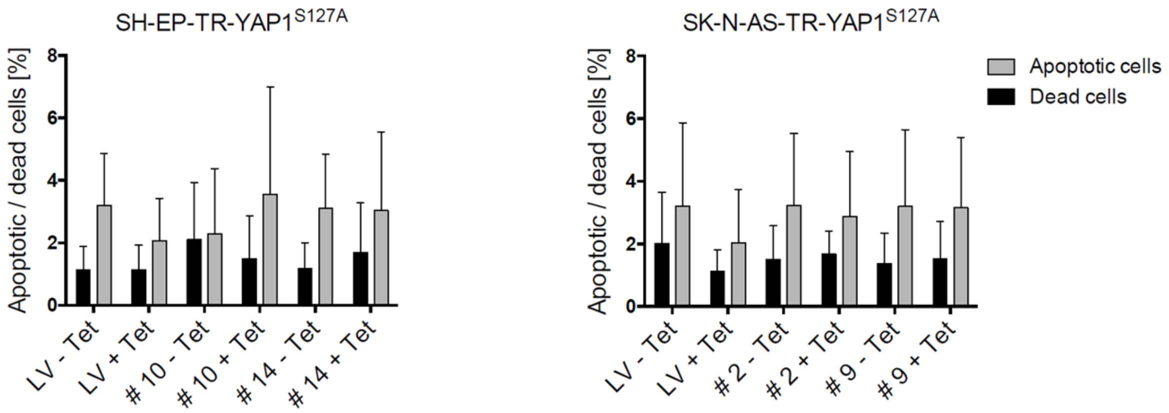

Supplementary Figure S8: YAP1 ^{S127A} activation in neuroblastoma cells only had a minor affect on apoptosis. Shown are different SH-EP-TR-YAP1^{S127A} and SK-N-AS-TR-YAP1^{S127A} clones (#) and empty vector controls (LV) after tetracycline (+Tet) or solvent (ethanol, -Tet) treatment for 72 hours. Flow cytometry analysis of YAP1-activated (+Tet) and ethanol-treated (-Tet) control cells for SH-EP-TR-YAP1^{S127A} (**left**) and SK-N-AS-TR-YAP1^{S127A} clones (**right**) assessing proportions of PI /Annexin V double-positive dead cells and Annexin V single-positive apoptotic cells. Bar plots represent mean ±SD of n=3. Statistical significance was assessed using the Mann-Whitney U test.

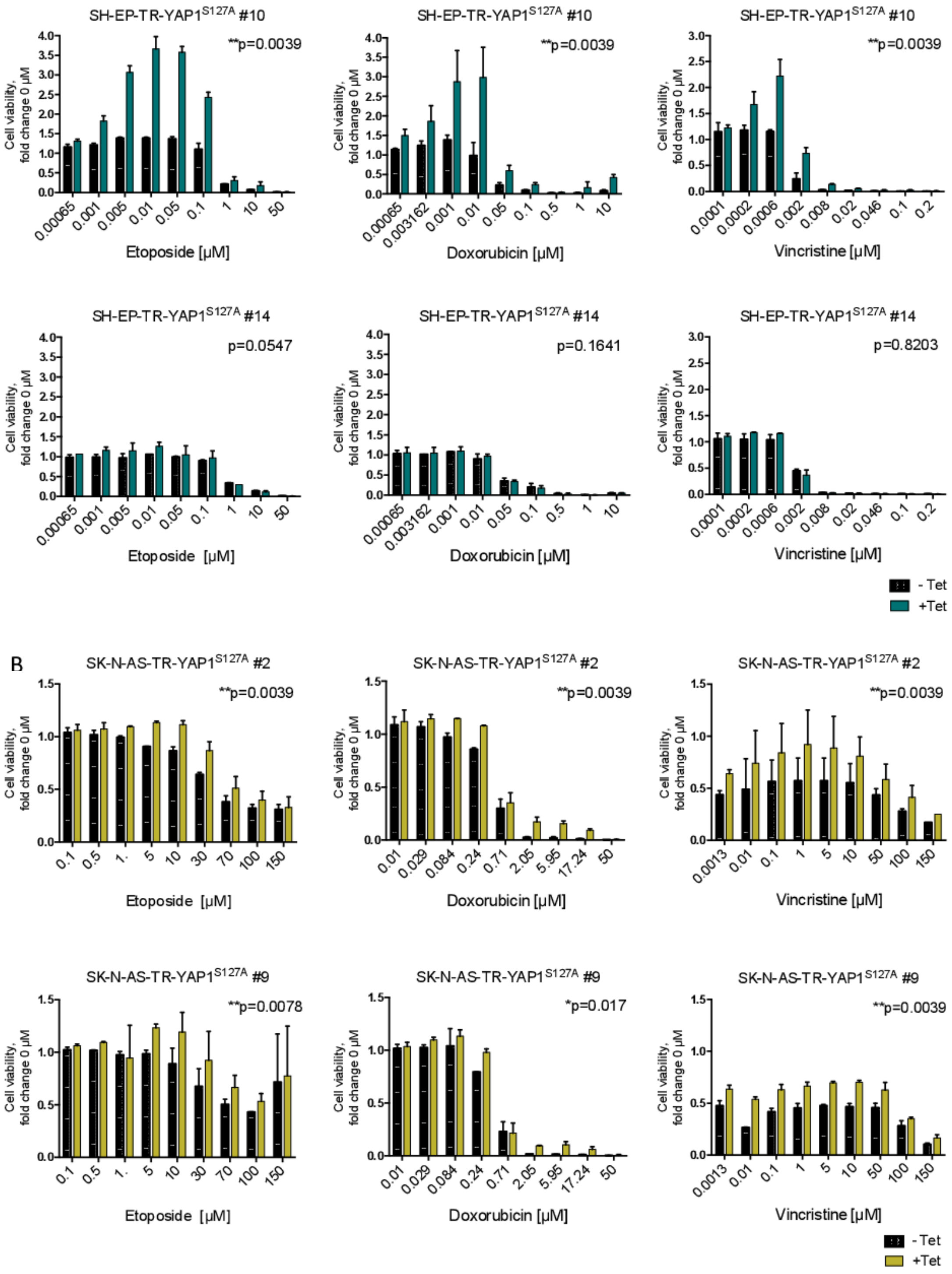

Supplementary Figure S9: Cell viability of YAP1-activated cells under treatment with etoposide, doxorubicin and vincristine. Two YAP1-inducible cell lines were treated with different concentrations of etoposide, doxorubicin and vincristine. SH-EP-TR-YAP1^{S127A} clone #10 viability was significantly higher under treatment of any single agent of the three when YAP1^{S127A} was activated (+Tet) compared to solvent control (-Tet, **blue top panel).** No difference was detected for clone #14. Both SK-N-AS TR-YAP1^{S127A} cell clones were more viable upon doxorubicin, etoposide or vincristine treatment, when YAP1^{S127A} was overexpressed (**yellow bottom panel**). Bars represent mean enrichment over the untreated controls (± SD, n=3). P-values were calculated using the Wilcoxon matched-pairs signed rank test. p*0.01 < p < 0.05, **0.001 < p < 0.01

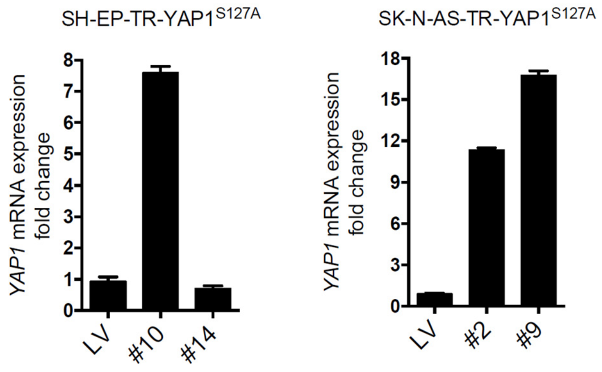

Supplementary Figure S10: Lost YAP1 activation in SH-EP-TR-YAP1^{S127A} clone #14. YAP1 gene expression level in induced SH-EP-TR-YAP1^{S127A} (**left**) and SK-N-AS-YAP1^{S127A} (**right**) cell clones are shown after treatment and simultaneous harvest with chemotherapeutically treated cells. Quantitative PCR detected no YAP overexpression in comparison to the empty vector control (LV) in clone #14. Bars represent expression enrichment in YAP1-activated cells over solvent controls (means ±SD) in relation to *SDHA* expression as a control for equivalent cell numbers.
