# Supplementary material for: YAP1 Enhances Mesenchymal-Type Gene Expression in Human Adrenergic-Type Neuroblastoma Cells
Source: Cancers (Basel). 2026 Jan 26;18(3):383. doi: 10.3390/cancers18030383 (PMC12897277; doi:10.3390/cancers18030383)
Supplement: Supplementary file 1 [file cancers-18-00383-s001.zip › NB-YAP1_Fig1B_Uncropped-Western-blots_15.12.2025.pptx]

## Slide 1
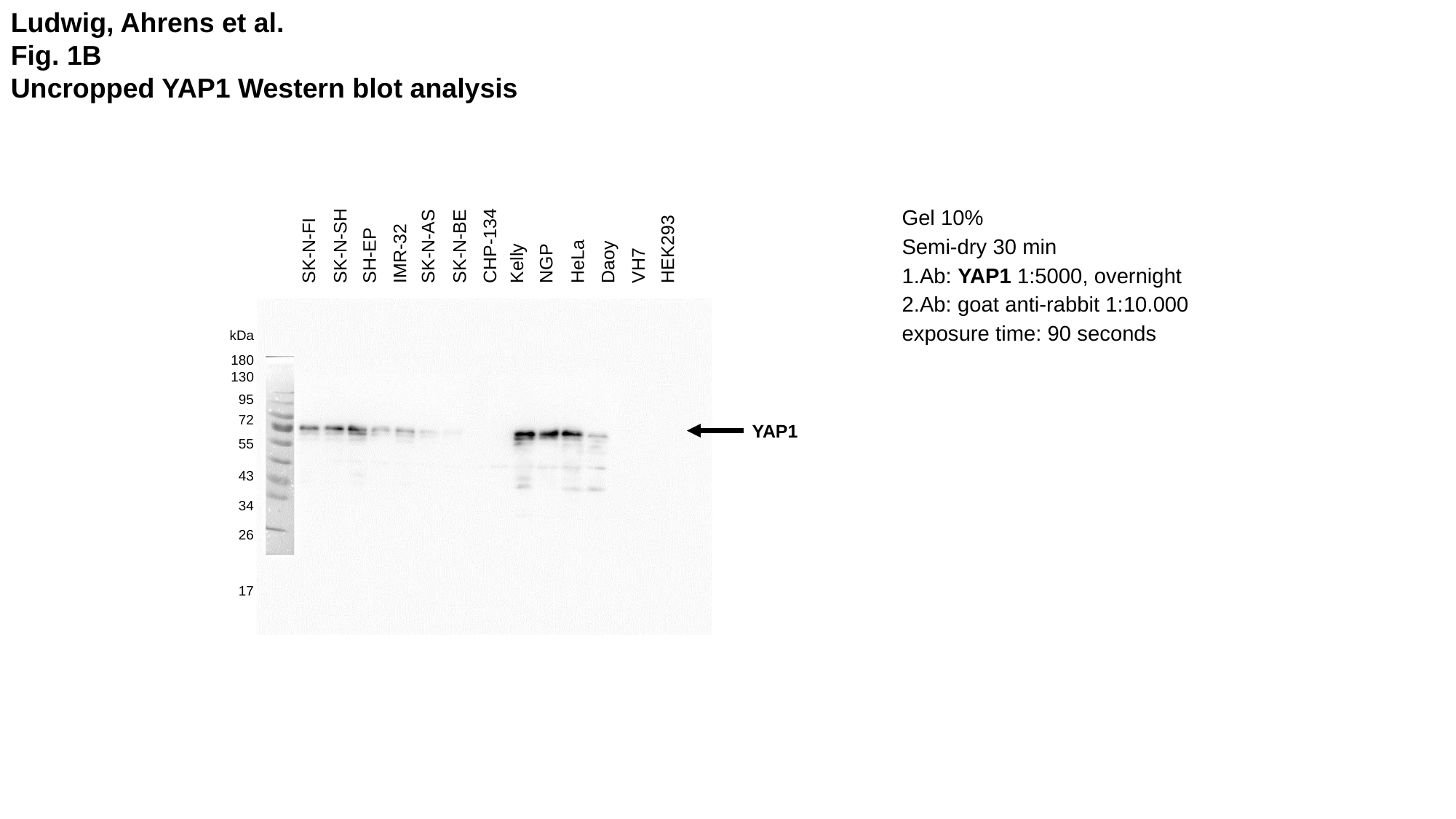

Ludwig, Ahrens et al.
Fig. 1B
Uncropped YAP1 Western blot analysis
Gel 10%
Semi-dry 30 min
1.Ab: YAP1 1:5000, overnight
2.Ab: goat anti-rabbit 1:10.000
exposure time: 90 seconds
SK-N-FI
SK-N-SH
SH-EP
IMR-32
SK-N-AS
SK-N-BE
CHP-134
Kelly
NGP
HeLa
Daoy
VH7
HEK293
kDa
180
130
95
72
YAP1
55
43
34
26
17

## Slide 2
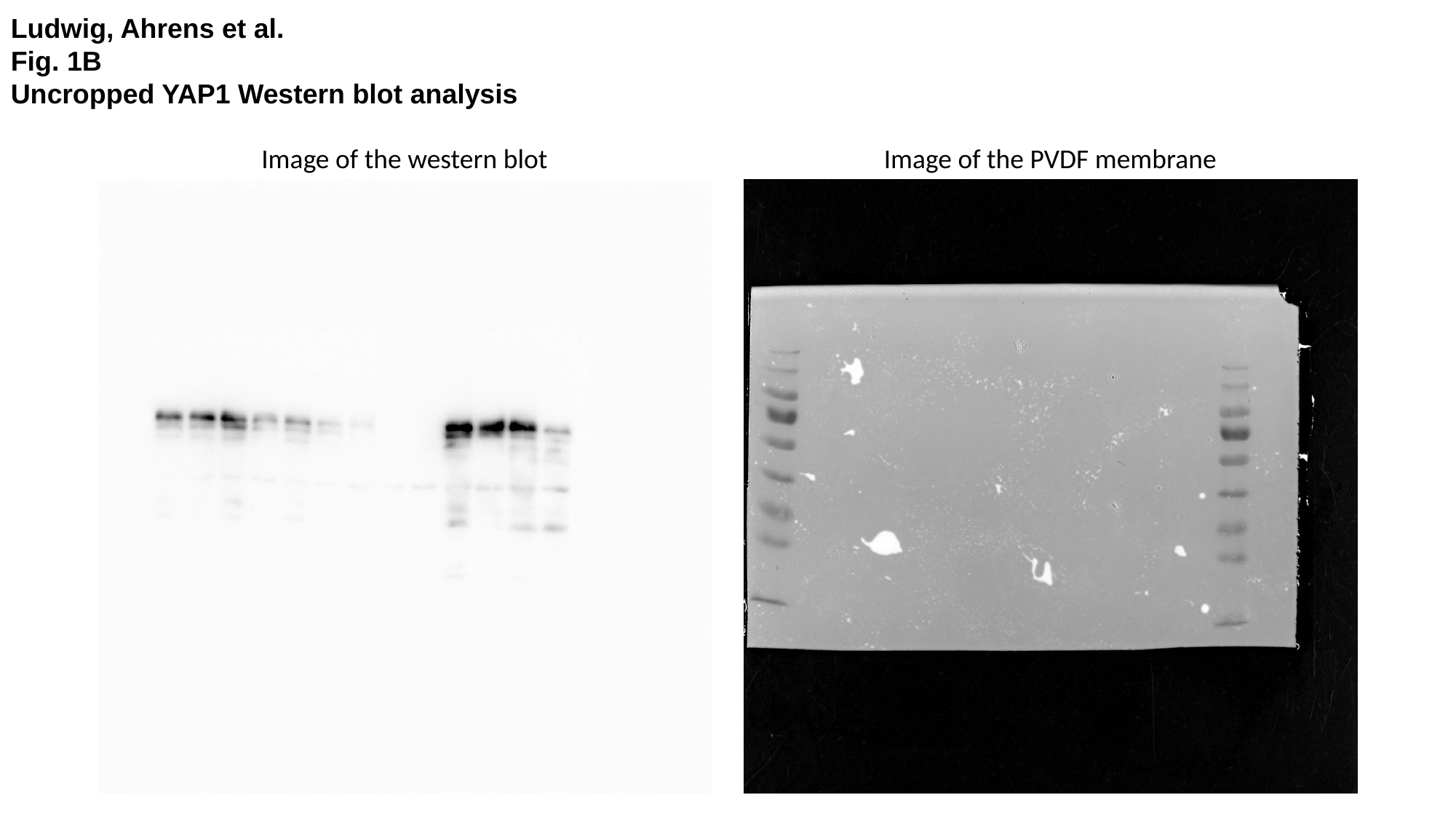

Ludwig, Ahrens et al.
Fig. 1B
Uncropped YAP1 Western blot analysis
Image of the western blot
Image of the PVDF membrane

## Slide 3
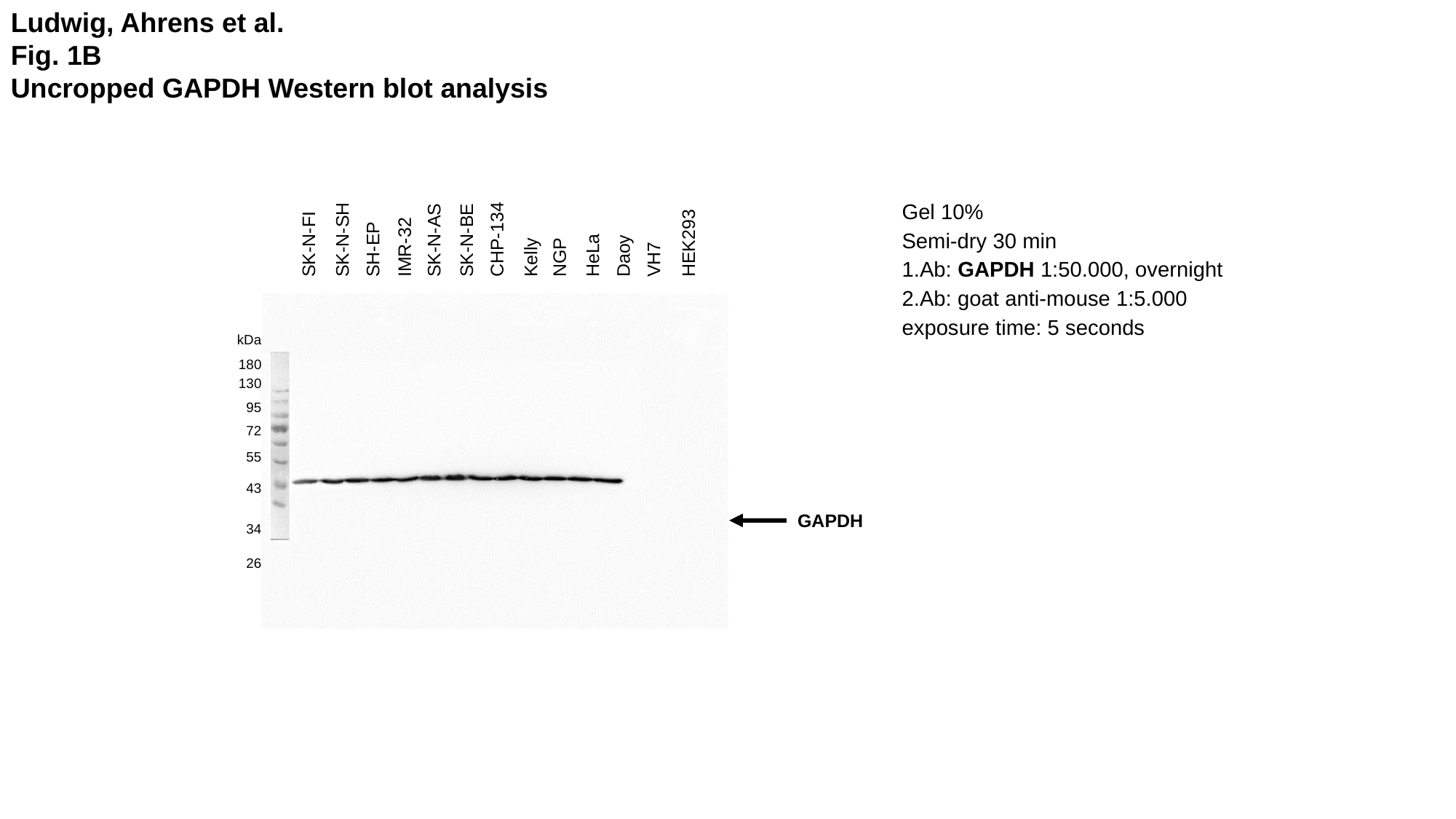

Ludwig, Ahrens et al.
Fig. 1B
Uncropped GAPDH Western blot analysis
Gel 10%
Semi-dry 30 min
1.Ab: GAPDH 1:50.000, overnight
2.Ab: goat anti-mouse 1:5.000
exposure time: 5 seconds
SK-N-FI
SK-N-SH
SH-EP
IMR-32
SK-N-AS
SK-N-BE
CHP-134
Kelly
NGP
HeLa
Daoy
VH7
HEK293
kDa
180
130
95
72
55
43
GAPDH
34
26

## Slide 4
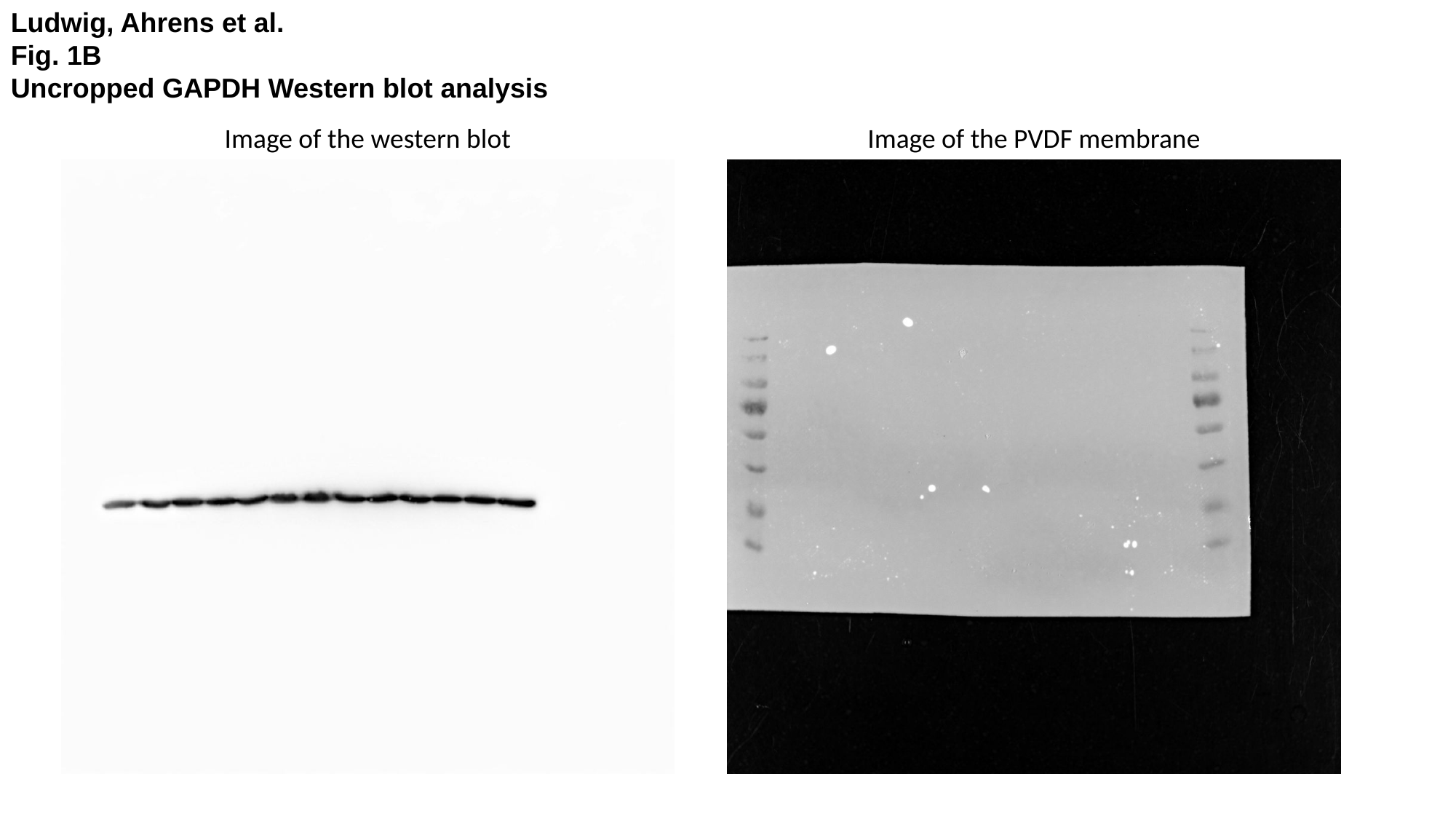

Ludwig, Ahrens et al.
Fig. 1B
Uncropped GAPDH Western blot analysis
Image of the western blot
Image of the PVDF membrane
