# Supplementary material for: YAP1 Enhances Mesenchymal-Type Gene Expression in Human Adrenergic-Type Neuroblastoma Cells
Source: Cancers (Basel). 2026 Jan 26;18(3):383. doi: 10.3390/cancers18030383 (PMC12897277; doi:10.3390/cancers18030383)
Supplement: Supplementary file 1 [file cancers-18-00383-s001.zip › NB-YAP1_Fig2A-B_Uncropped-Western-blots_15.12.2025.pptx]

## Slide 1
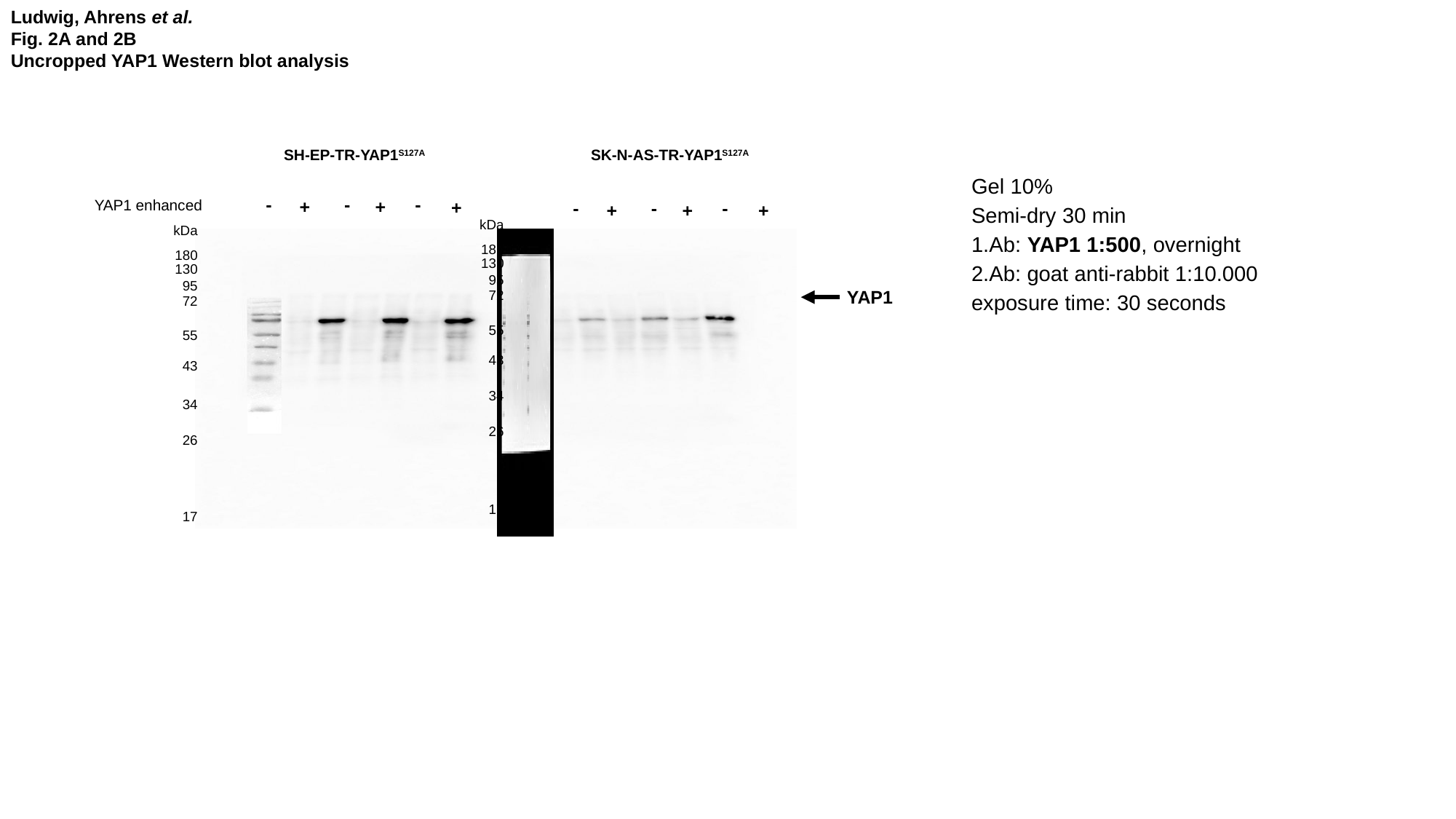

Ludwig, Ahrens et al.
Fig. 2A and 2B
Uncropped YAP1 Western blot analysis
SH-EP-TR-YAP1S127A
SK-N-AS-TR-YAP1S127A
Gel 10%
Semi-dry 30 min
1.Ab: YAP1 1:500, overnight
2.Ab: goat anti-rabbit 1:10.000
exposure time: 30 seconds
-
-
-
YAP1 enhanced
+
+
+
-
-
-
+
+
+
kDa
kDa
180
180
130
130
95
95
YAP1
72
72
55
55
43
43
34
34
26
26
17
17

## Slide 2
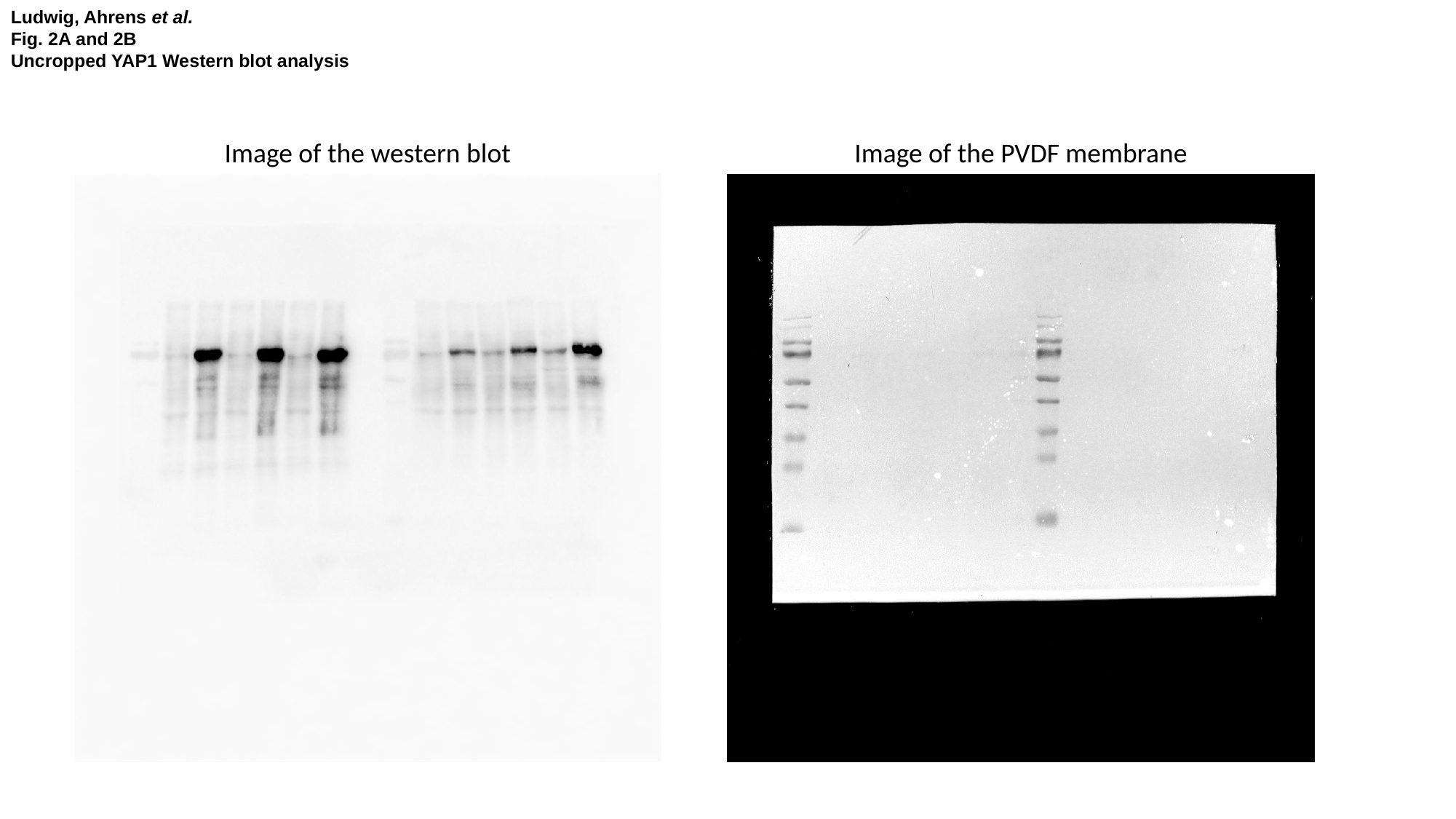

Ludwig, Ahrens et al.
Fig. 2A and 2B
Uncropped YAP1 Western blot analysis
Image of the western blot
Image of the PVDF membrane

## Slide 3
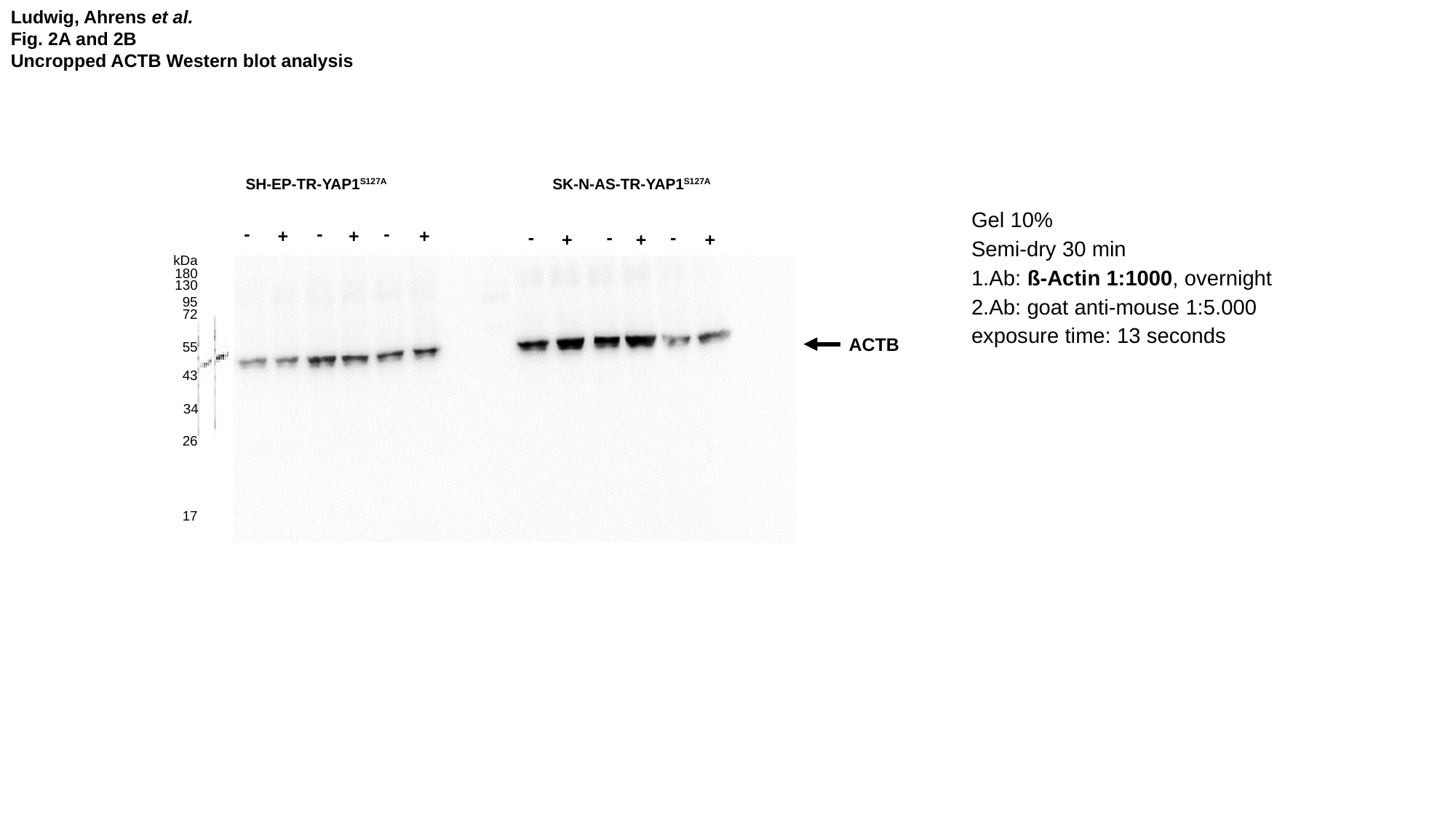

Ludwig, Ahrens et al.
Fig. 2A and 2B
Uncropped ACTB Western blot analysis
SH-EP-TR-YAP1S127A
SK-N-AS-TR-YAP1S127A
Gel 10%
Semi-dry 30 min
1.Ab: ß-Actin 1:1000, overnight
2.Ab: goat anti-mouse 1:5.000
exposure time: 13 seconds
-
-
-
+
+
+
-
-
-
+
+
+
kDa
180
130
95
72
ACTB
55
43
34
26
17

## Slide 4
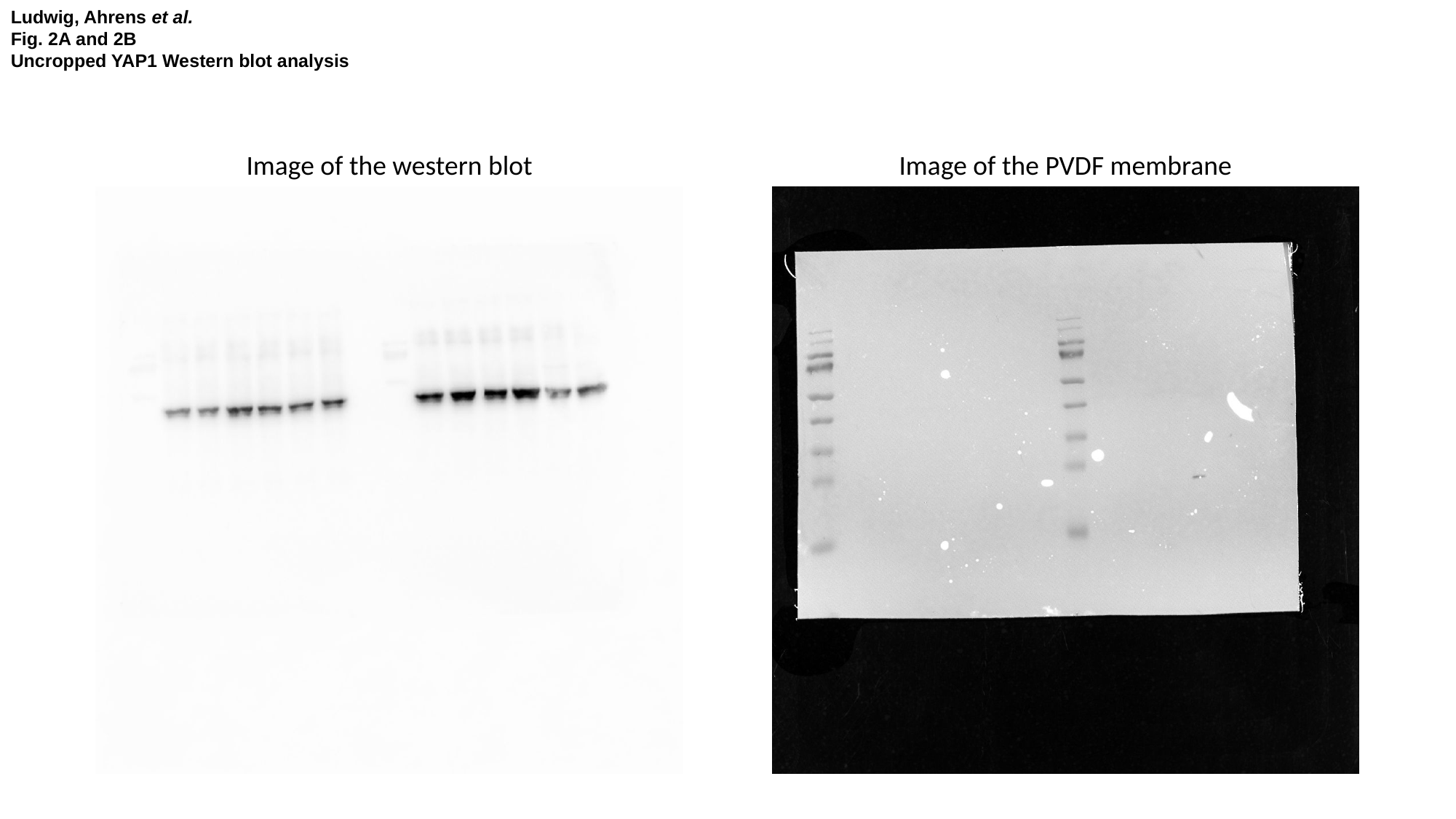

Ludwig, Ahrens et al.
Fig. 2A and 2B
Uncropped YAP1 Western blot analysis
Image of the western blot
Image of the PVDF membrane
